# The *Malus domestica* sugar transporter gene family: identifications based on genome and expression profiling related to the accumulation of fruit sugars

**DOI:** 10.3389/fpls.2014.00569

**Published:** 2014-11-05

**Authors:** Xiaoyu Wei, Fengli Liu, Cheng Chen, Fengwang Ma, Mingjun Li

**Affiliations:** State Key Laboratory of Crop Stress Biology for Arid Areas, College of Horticulture, Northwest A&F UniversityYangling, China

**Keywords:** *Malus*, fruit sugar, EDR6, SWEET, genome, sugar transporter

## Abstract

In plants, sugar transporters are involved not only in long-distance transport, but also in sugar accumulations in sink cells. To identify members of sugar transporter gene families and to analyze their function in fruit sugar accumulation, we conducted a phylogenetic analysis of the *Malus domestica* genome. Expression profiling was performed with shoot tips, mature leaves, and developed fruit of “Gala” apple. Genes for sugar alcohol [including 17 sorbitol transporters (SOTs)], sucrose, and monosaccharide transporters, plus SWEET genes, were selected as candidates in 31, 9, 50, and 27 loci, respectively, of the genome. The monosaccharide transporter family appears to include five subfamilies (30 MdHTs, 8 MdEDR6s, 5 MdTMTs, 3 MdvGTs, and 4 MdpGLTs). Phylogenetic analysis of the protein sequences indicated that orthologs exist among *Malus, Vitis*, and *Arabidopsis*. Investigations of transcripts revealed that 68 candidate transporters are expressed in apple, albeit to different extents. Here, we discuss their possible roles based on the relationship between their levels of expression and sugar concentrations. The high accumulation of fructose in apple fruit is possibly linked to the coordination and cooperation between *MdTMT1/2* and *MdEDR6*. By contrast, these fruits show low *MdSWEET4.1* expression and a high flux of fructose produced from sorbitol. Our study provides an exhaustive survey of sugar transporter genes and demonstrates that sugar transporter gene families in *M. domestica* are comparable to those in other species. Expression profiling of these transporters will likely contribute to improving our understanding of their physiological functions in fruit formation and the development of sweetness properties.

## Introduction

In plants, soluble sugars [i.e., sucrose (Suc), monosaccharides, and polyols] are essential molecules that not only provide energy and building blocks for growth and development, but also constitute osmotic, nutrient, and signal molecules (Ruan, 2014). In fruit crops, soluble sugars are also central to quality; their accumulation during the maturation process largely determines sweetness at harvest.

**Figure 1:**
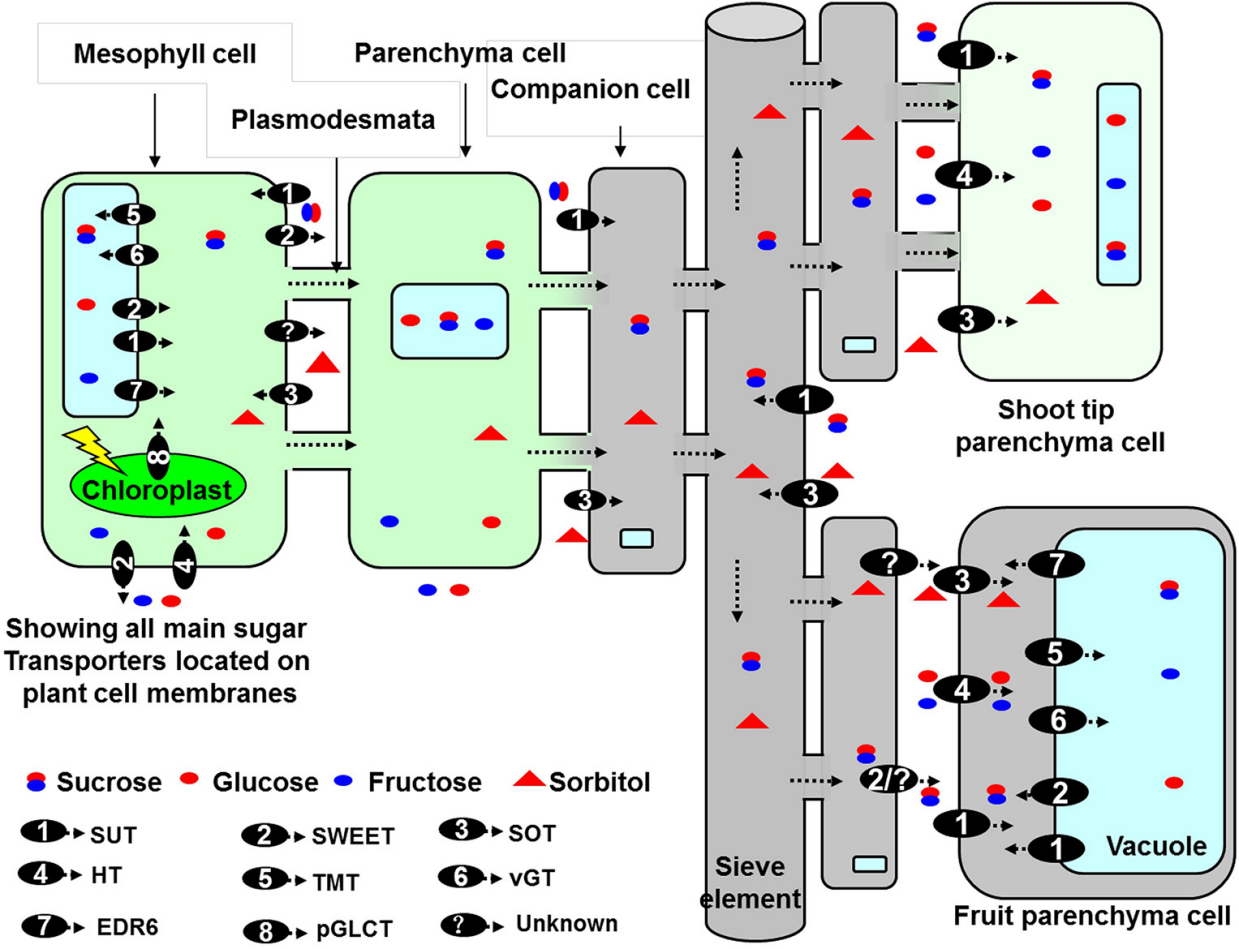
**Model for sugar transport in apple (Zhang et al., 2004; Doidy et al., 2012; Li et al., 2012; Chardon et al., 2013; Ruan, 2014)**. Sorbitol (Sor) and sucrose (Suc), both of which are synthesized in mesophyll cell of source leaves, are loaded into phloem via symplast pathway. After translocating to SE-CC of fruit, both are unloaded into fruit parenchyma cells via apoplast pathway. In apple cells, four families of transporters are implicated in the distribution of Suc, Sor, fructose (Fru), and glucose (Glc): sucrose transporter (SUT), SWEET, sorbitol transporter (SOT), and monosaccharide transporter (MST) [including hexose transporter (HT), tonoplast membrane transporter (TMT), vacuolar glucose transporter (vGT), ERD six-like transporters (EDR6), and plastid glucose transporters (pGLCT)]. At the plasma membrane, all of SUTs, SOTs, and HTs have been characterized as H+/sugar importers, and transport influx of extracellular Suc, Sor, and hexose. By contrast, SWEETs function as energy-independent uniporters that mainly mediate sugar efflux. At the vacuolar membrane, vGTs, and TMTs function as sugar/H+ antiporters transporting sugars into the vacuole, while EDR6 and SWEET IV subfamilies are involved in energy-independent sugar efflux from the vacuole. Especially, in fruit cells these special tonoplast transporters may be involved in accumulating Fru, Glc, and Suc in vacuole. Additionally, pGLCTs may also play a role in efflux of sugars from plastids.

In multicellular organisms, the movement of sugar is an essential part of long-distance transport for assimilates from source to sink organs, storage of carbon, regulation of osmotic potential and turgor, and the cellular exchange of carbon and energy (Slewinski, 2011). Biochemical and molecular researchers have argued that hexoses or sucrose are transported into the chloroplast (Weber et al., 2000), the vacuoles (Martinoia et al., 2000), and the Golgi apparatus (Wang et al., 2006). As relatively large and polar solutes, soluble sugars require proteins to facilitate efficient diffusion across membranes. Not only the loading and the unloading of the conducting complex, but also the allocation of sugars into source and sink cells, are controlled by sugar transporters that mediate the movement of Suc (sucrose transporter: SUT) (Ruan, 2014), reducing monosaccharides (monosaccharide transporter or hexose transporter: MST or HT) (Büttner, 2010; Slewinski, 2011), or sugar alcohols [sorbitol (Sor), mannitol, xylitol, et al.] (Noiraud et al., 2001; Gao et al., 2005; Fan et al., 2009). This model is depicted in Figure 1.

Efficient movement of sugars across membranes requires the operation of multiple transporters, which will have different energetic and kinetic properties suited for efficient unloading into cell wall spaces, uptake of Suc or other sugars leaked or locally transported via the apoplasm, loading from the cytosol into storage vacuoles, and the fine-tuning of sugar fluxes for homoeostasis and interactions with other proteins for sugar sensing and signaling (Slewinski, 2011; Ruan, 2014). To achieve this, multiple families for genes encode these transporters.

Since the cloning of the first MST (Sauer and Tanner, 1989), SUT (Riesmeier et al., 1992), and polyol transporter in plants (Noiraud et al., 2001), many genes in those families have been isolated from various species. For example, the complete *Arabidopsis* genome contains nine SUT-like sequences (Ruan, 2014) plus a monosaccharide transporter (-like) gene family that has 53 members in seven subfamilies (Büttner, 2010). The *Vitis vinifera* (grapevine) genome has four SUTs and 59 MSTs (Afoufa-Bastien et al., 2010). Evolutionary analysis of plant MSTs has revealed seven ancient subfamilies in land plants (Slewinski, 2011). Recently identified SWEET proteins in a distinct transporter family account for 17 members in *Arabidopsis* and 21 in rice. These members can transport Suc or glucose (Glc) (Chen et al., 2010, 2012) or fructose (Fru) (Chardon et al., 2013; Klemens et al., 2013), and are involved in loading (Chen et al., 2012), sugar storage (Chardon et al., 2013), nectar production (Lin et al., 2014), and interactions between plants and fungi (Chen et al., 2010).

Knowledge is gradually increasing about the intracellular distribution of sugar transporters and their roles in regulating this transport, signaling, and homeostasis in model herbaceous plants, e.g., *Arabidopsis*. Three families of transporters—SUTs, MSTs, and SWEETs—are mainly implicated in the distribution of sugars within most plant cells (reviewed by Doidy et al., 2012). At the plasma membrane, most transporters have been characterized as H+/sugar importers. However, *ZmSUT1* (Carpaneto et al., 2005) and *AtSUC4* (Schneider et al., 2011) also mediate the active efflux of Suc. By contrast, SWEETs function as energy-independent uniporters that mediate sugar influx and/or efflux (Chen et al., 2010). Both *AtSWEET11*and *12* have been localized to the plasma membrane (Chen et al., 2012) whereas *AtSWEET17* occurs in the tonoplast membrane, where it transports Fru (Chardon et al., 2013). At the vacuolar membrane, the MST subfamilies, vacuolar glucose transporter (vGT), and tonoplast membrane transporter (TMT) function as sugar/H+ antiporters that load sugars into the vacuole (Wormit et al., 2006; Aluri and Büttner, 2007; Schulz et al., 2011). Proteins of the MST subfamily of ERD six-like transporters (ERD6 or ESL1) are likely involved in energy-independent sugar efflux from the vacuole (Poschet et al., 2011; Klemens et al., 2014).

Research data have also suggested that expression of sugar transporters might be regulated at the transcriptional level by distinct but usually converging signaling pathways that depend upon either developmental and environmental cues or metabolic and hormonal signals. Despite the progress made in identifying genes that encode sugar transporters, little is known about the roles and transcriptional regulation of these genes, especially in crop plants. It is unknown how different transporter orthologs modulate sugar distribution and homeostasis in plant cells, and how they control sugar accumulations in storage tissues and cells. Therefore, analysis of these orthologs in different species might help improve our understanding of their biological functions.

Apple (*Malus domestica* Borkh.), a member of the *Rosaceae* family, is among the most important commercial fruit crops grown worldwide. Apple and other *Rosaceae* tree fruits synthesize sorbitol (Sor) and Suc in source leaves. Both are then translocated to and utilized in fruit, with Sor accounting for approximately 60–70% of the photosynthates produced in the leaves. They are loaded via the symplasmic pathway for transport in the phloem (Reidel et al., 2009). After being unloaded from SE-CC (sieve elements and companying cells) complexes into the cell wall space of apple fruit (Zhang et al., 2004), Sor is taken up into the cytosol of parenchyma cells by a sorbitol transporter (SOT) located on the plasma membrane. Meanwhile, Suc is directly transported into parenchyma cells by SUT on the plasma membrane, or else first converted to Fru and Glc by cell wall invertase and then transported into parenchyma cells by hexose transporters (HT) (Figure 1; Zhang et al., 2004; Fan et al., 2009; Li et al., 2012). In the cytoplasm of the mesocarp cells, sucrose and hexoses must be transported into the vacuole via tonoplastic transporters (Figure 1). Compared with sink organs in model plants that import and metabolize only sucrose (e.g., *Arabidopsis, Solanum tuberosum*, and *Populus*), apple is unique in its metabolism and accumulation of sugars. More than 80% of the total carbon flux goes through fructose (because almost all of the Sor and half of the Suc are converted to Fru) (Li et al., 2012). Consequently, the characteristics of sugar transporters can differ between apple and other plants. Roles in unloading and changes in expression during fruit development have been preliminarily reported for *MdSOT* (Gao et al., 2005) and *MdSUT1* (Fan et al., 2009), and Li et al. (2012) identified the some members of gene families encoding transporters (including MdSUT, MdTMT, and MdvGT) and analyzed the relationship of their transcripts with sugar accumulation during fruit development of “Greensleeves” apple, but there was no exhaustive knowledge on apple sugar transporter based on genome, especially for MST and SWEET families. Identification and characterization of these transporter genes in *Malus* are important steps in understanding the roles of these proteins in growth and development as well as the process of sugar accumulation in the fruit.

Here, we identified *SUT, MST*, and *SWEET* genes in the *M. domestica* genome (Velasco et al., 2010) through phylogenetic analysis and compared them primarily with *Arabidopsis* transporters. Real-time PCR was used to determine expression patterns in different tissue types, and to examine the relationship between relative transcript abundances and sugar accumulation over time. Our objective was to devise a useful approach for investigating the function of sugar transporters and the development of sweetness traits in apple fruit.

## Materials and methods

### Plant materials

Nine-year-old “Gala” apple trees (*Malus domestica*) grafted onto rootstock *M*. *sieversii* were trained as a central leader system and grown at the spacing of 3 (row) × 4 (interval) m in North-South rows in an experimental orchard at the Horticultural Experimental Station of Northwest A & F University, Yangling, China. Fungicides and pesticides were sprayed at regular intervals throughout the growing season to protect the plants from diseases and insects. At 16, 34, 55, 75, 98, and 122 days after bloom (DAB), fruits were sampled from the south side of the tree canopy between 3:00 and 4:00 p.m., under full sun exposure. On each collection date, six apples were from 3 trees and pooled for one replicate, with five replications in all from in total 15 trees. The fruits were immediately weighed, cut into small pieces after removing the core, and frozen on-site in liquid nitrogen (2-min interval between harvest and freezing). To compare the expression patterns of related genes in source and sink tissues, we also collected mature leaves and shoot tips at 34 DAB. All frozen samples were stored at −80°C.

### Identification of candidate genes

Candidate genes were identified by performing a BlastP analysis against the apple gene set (amino acids) in the *Malus* Genome Database (http://www.rosaceae.org) (Velasco et al., 2010) from the “Fondazione Edmund Mach Istituto Agrario San Michele All'Adige,” Italy, or IASMA (http://www.rosaceae.org/tools/ncbi_blast). As query, we used sequences for *Arabidopsis thaliana* polyol/monosaccharide transporter (PMT) and sugar transporters [including STP (sugar transport protein) or HT, EDR6 (early-responsive to dehydration), TMT, vGT, pGLCT (plastid glucose transporter), and SWEET family members] that were obtained from The *Arabidopsis* Information Resource (http://arabidopsis.org/). Additionally, reported *MdSOTs* (Li et al., 2011) and *MdSUT* (Fan et al., 2009) were also employed as query. An *E*-value of 1.00E-10 was set as the threshold. Putative candidate gene sequences were retrieved from the *Malus* Genome Database (http://www.rosaceae.org/species/malus/malus_x_domestica/genome_v1.0). To confirm their expression in apple and their reliability as putative candidates, we used their corresponding sequences in a Blast search against the *Malus* EST database at the National Center for Biotechnology Information (http://www.ncbi.nlm.nih.gov/). A predicted gene was considered expressed when ESTs in the *Malus* transcriptome showed high similarity, i.e., length >300 bp and identity score >98%, were identified. After comparing between each predicted gene and its EST-constructed contig or EST, we re-conducted the Blast analysis with divergent genes against all predictions in apple (nucleic acids) (http://www.rosaceae.org/tools/ncbi_blast). Here, EST-constructed contigs or ESTs were used so that a sequence concordant with the EST would be found in all predictions.

### Sequence similarities and phylogeny analyses

Multiple alignments of the transporter proteins were obtained using DNAMAN software. To construct the phylogenetic tree, we downloaded the full-length amino acid sequences of *PMT*, sugar transporters, and SWEET family sequences from *Arabidopsis* and grapevine in the NCBI protein database (http://www.ncbi.nlm.nih.gov/guide/). Those sequences were then aligned with the integrated MUSCLE alignment program in MEGA5 (Molecular Evolutionary Genetics Analysis) with default parameters. Phylogenetic analysis was performed via the Neighbor-Joining method, using MEGA5 software and bootstrap tests replicated 1000 times. In addition, the subcellular localizations of candidate genes were predicted with TargetP software (http://www.cbs.dtu.dk/services/TargetP) and the WoLF PSORT version of PSORT II (http://wolfpsort.org/).

Information about chromosome lengths and gene locations have previously been reported for the apple genome (Velasco et al., 2010). Locations and transcriptional directions were assigned to the chromosomes by MapDraw software (Liu and Meng, 2003).

### Analysis of mRNA expression

Quantitative reverse transcription-polymerase chain reaction (qRT-PCR) was used to analyze the expression of all selected genes (Supplementary Data Excel Files1–4). Total RNA was extracted from samples by the modified CTAB method (Gasic et al., 2004), and DNase was used to remove DNA before reverse-transcription. After sequence similarities were examined, gene-specific primers (Supplementary Data Excel Files 5) were designed from the coding sequences of apple genes, using Primer5 software. Primer specificity was determined by RT-PCR and Melt Curve analysis. Afterward, qRT-PCR was performed with an iScript cDNA Synthesis Kit (Bio-Rad, Hercules, CA, USA) according to the manufacturer's protocol. Amplified PCR products were quantified by an iQ5 Multicolor Real-Time PCR Detection System (Bio-Rad) and an iQ SYBR Green Supermix kit (Bio-Rad). *Actin* (CN938023) transcripts were used to standardize the different cDNA samples throughout the test (Li et al., 2012). For all samples, total RNA was extracted into five tubes from five replicates and then mixed in a tube used for reverse-transcription. The qRT-PCR experiments were done with three technical replicates. Data were analyzed by the ddCT method in iQ5 2.0 standard optical system analysis software.

### Measurements of soluble sugars

Soluble sugars and hexose phosphates were obtained and derivatized as described by Wang et al. (2010). Briefly, samples (0.09–0.11 g) were extracted in 1.4 mL of 75% methanol, with ribitol added as the internal standard. After the non-polar metabolites were fractionated into chloroform, 2 μl of the polar phase was transferred into 2.0-mL Eppendorf vials for measurements of the metabolites (Sor, Fru, Glc, Suc, galactose: Gal and myo-inositol) of each sample. They were dried under vacuum without heating and then derivatized sequentially with methoxyamine hydrochloride and N-methyl-N-trimethylsilyl-trifluoroacetamide (Lisec et al., 2006). Afterward, the metabolites were analyzed with a Shimadzu GCMS-2010SE (Shimadzu Corporation, Tyoto, Japan). These metabolites were identified by comparing their fragmentation patterns with those from a mass spectral library generated on our GC/MS system, and from an annotated quadrupole GC–MS spectral library downloaded from the Golm Metabolome Database (http://csbdb.mpimp-golm. mpg.de/csbdb/gmd/msri/gmd_msri.html. Quantifications were based on standard curves generated for each metabolite and internal standard.

## Results

### Sorbitol transporters in the *malus domestica* genome

**Figure 2:**
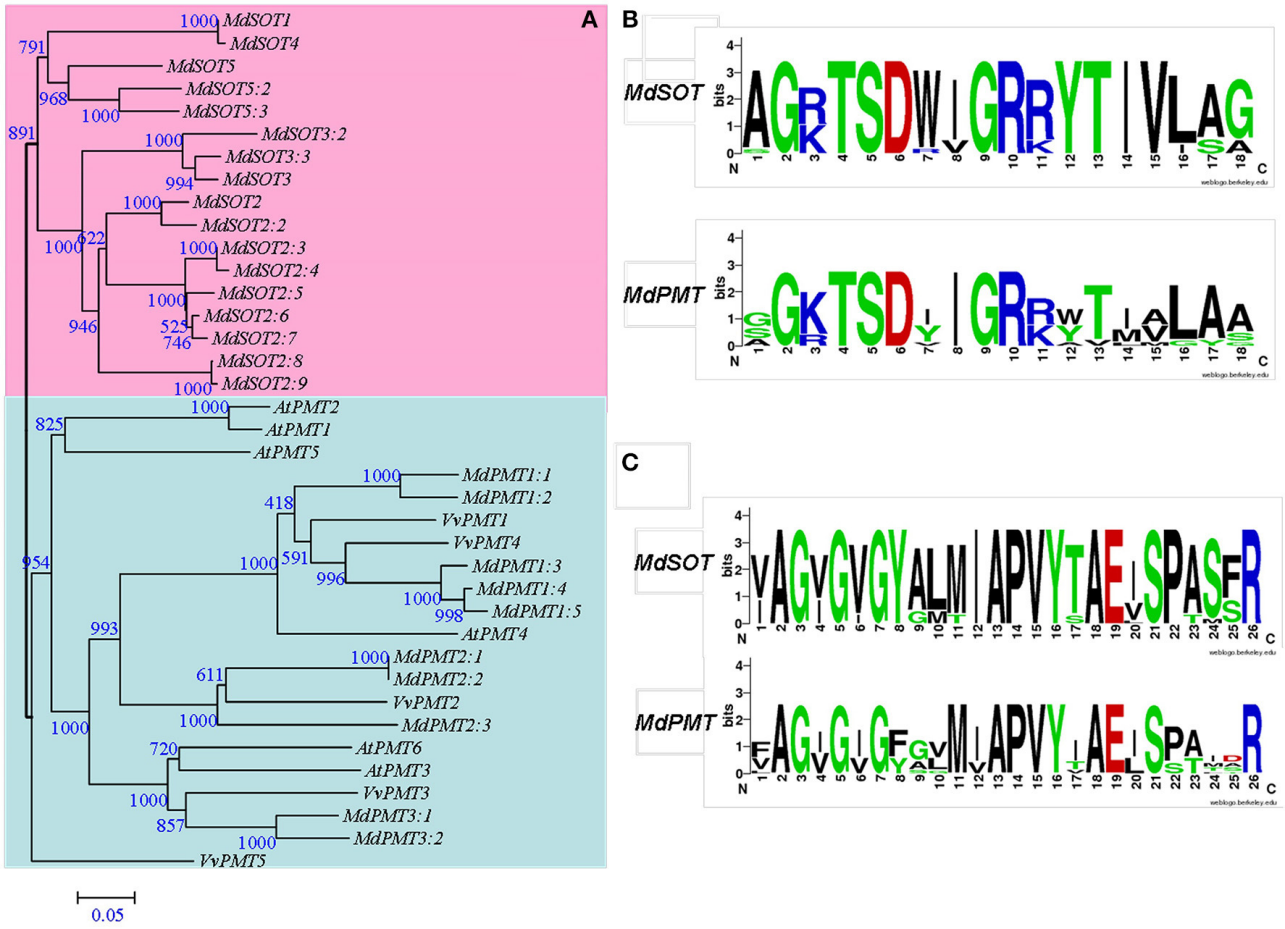
**Phylogenetic analysis (A) of *Malus domestica* polyol transporter families with *Vitis vinifera* and *Arabidopsis thaliana*, and conserved properties of transporter Motif 1 (B) and 2 (C) in *MdPMT* and *MdSOT* subfamily**. Tree was constructed via Neighbor-Joining method with 1000 bootstrap replications. Accession numbers for all *Malus* genes are listed in Supplementary Data Excel File 1. Accession numbers for *Vitis* genes were reported by Afoufa-Bastien et al. (2010). Accession numbers for *A. thaliana*: AT2G16120.1/*ATPMT1*, AT2G16130.1/*ATPMT2*, AT2G20780.1/*ATPMT4*, AT2G18480.1/*AtPMT3*, AT3G18830.1/*ATPMT5*, and AT4G36670.1/*ATPMT6*.

Using *AtPMT* and reported *MdSOT* sequences as queries, we conducted a BlastP search to identify candidate genes in *Malus*. In all, 40 consensus gene sets containing major facilitator superfamily (MFS) domains were selected from the “Gold Delicious” apple genome database, with an EXP cutoff <1.00E-10. Based on pair-wise comparisons of these predicted genes against *Vitis vinifera* and peach genome databases, as well as the TrEMBL database, all genes were highly similar to orthologs in *Vitis* and peach. In addition, 30 selected genes showed very high homology with reported *MdSOT* (Supplementary Data Excel File 1—Sheet 1). To confirm their expression in apple and to examine the reliability of putative candidate gene sequences, we used the corresponding sequences of candidate genes for a BLAST search against the *Malus* EST database. Sixteen predicted genes (Supplementary Data Excel File 1—Sheet 2) had high similarities in EST sequences (score > 300 bp, identity > 98%). However, the sequences of six selected genes diverged from their corresponding EST-constructed contigs. A concordant sequence with an EST was found in all predictions from either the *Malus* genome database or GenBank. For *MdSOT1*, −4, and −5, we were unable to find concordant sequences in all predicted genes. Based on reports of functional *PMT*s and *SOT*s, we set thresholds for the number of transmembrane domains(≥8) and the length of coding amino acids (>300 a.a.). After re-screening, we selected 27 genes as possible members of the *MdSOT* or *MdPMT* family. All candidates from the *AtPMT* families were aligned to elucidate their evolutionary relationships, and were named according to our phylogenetic tree (Figure 2; Supplementary Data Excel File 1).

This phylogenetic tree revealed two distinct groups, with the first containing all reported *MdSOT*s, but no *PMT*s from *Arabidopsis* or *Vitis*. In this group, *MdSOT1* had the highest similarity with *MdSOT4* and shared the same clade with *MdSOT5*. The apple genome showed two newly identified members (*MdSOT5.2* and −5.3) with high similarity to *MdSOT5*. *MdSOT3* was very similar to *MdSOT3.2* and −3.3 within a small, discrete clade. Eight genes shared high similarity with the amino acid sequence of *MdSOT2*, clustering in the same clade with *MdSOT3*. These included *MdSOT2.8* and−2.9, both of which had 99.4% similarity.

In the second group, 10 candidate genes were clustered with the *PMT*s of *Arabidopsis* and *Vitis*. The*MdPMT1* subfamily contained five members that had high homology and were in the same clade as *AtPMT4, VvPMT1*, and *VvPMT4*. In addition, the*MdPMT2* subfamily had three members that were orthologs of *VvPMT2*. Both *MdPMT3.1* and −3.2 had high similarity with *AtPMT3, AtPMT6*, and *VvPMT3*, and shared the same clade (Figure 2).

The approximate gene positions and transcriptional directions of candidate genes were marked on the physical map of chromosomes. In all, 17*MdSOT*s were located on four apple chromosomes (chr1, 3, 12, and 17). They included 11 on chr12 (including six in the *MdSOT2* subfamily) (Supplementary Figure 1) plus three on chr17. On chr12, 10 *MdSOT*s were in tandem at approximately 449 kb. From the *MdPMT* subfamilies, two pairs of *MdPMTs* showed tandem duplication, including *MdPMT2.1-MdPMT2.2* on chr2 and *MdPMT1.4-MdPMT1.5* on chr9. *MdPMT2.3* was located on chr15 while single *MdPMT1* members were found on chr5, 10, and 17, and two *MdPMT3* members occurred on chr2 and chr15.

### Sucrose transporters in the *malus domestica* genome

Using *AtSUT* or *AtSUC* protein sequences as query, we found 15 candidate gene sets in the *Malus* genome, including five predicted genes with high concordant EST sequences. Two sets were divergent with their corresponding ESTs; concordant sequences with ESTs were found in all predictions from the *Malus* database (Supplementary Data Excel File 2). Based on the reported functionality of SUT or SUC genes, we set the thresholds at ≥6 for the number of transmembrane domains and >200 a.a. for the length of coding amino acids. Nine genes were selected as candidates for the *MdSUT* family, including five *MdSUT*s we had described previously (Li et al., 2012).

Phylogenetic analysis divided the nine candidate *MdSUT*s into three groups that incorporated MdSUT1, MdSUT2, and MdSUT4 clades (Supplementary Figure 2; Supplementary Data Excel File 2). Three members of the *MdSUT1* group shared the same clade with *VvSUC27* but did not cluster in the same clade with those in the *AtSUT1* group (including *AtSUC1, AtSUC2*, and *AtSUC9*) that show high-affinity uptake in dicot plants (Ayre, 2011). *MdSUT2.1* and −2.2 were 93.6% similar and were clustered in the *SUT2* clade with *AtSUT2/SUC3* and *VvSUT2*. The *SUT4* clade contained four *MdSUT4* members that were similar to*AtSUC4* and *VvSUC1*.

The physical map presented nine *MdSUT*s on seven apple chromosomes. These included three *MdSUT4* members with tandem duplications at the end of chr8, plus *MdSUT4.1* on chr15 (Figure S1). Single members of the *MdSUT1* subfamily were located on chr10, chr9, and chr5, while *MdSUT2.1* and −2.2 were found on chr16 and chr13, respectively (Figure S1).

### Monosaccharide transportersin the *malus domestica* genome

We found 114 MST candidate gene sets in the *Malus* genome when *AtHT, AtSTP, AtEDR6, AtTMT*, or other sugar transporter protein sequences were used as query (Supplementary Data Excel File 3). Of these, 27 predicted genes had high-concordance EST sequences in the *Malus* EST database. Although five sets were divergent with the corresponding ESTs, concordant sequences with ESTs were found in all predictions of the *Malus* genome database. Simultaneously, 12 sets matched more reasonable splicing sequences in all predictions of that database. Based on the reported functionality of *AtSTP, AtHT*, and other genes, we set the threshold at >6 for the number of transmembrane domains and at >800 bp for the length of coding amino acids. Ultimately, 30 genes were selected as candidates for the *MdHT* family, including eight *MdEDR6*s, five *MdTMT*s, three *MdvGT*s, and three *MdpGLT*s. All candidates that were homologous to family members in the *A. thaliana* or *V. vinifera* genomes were aligned to elucidate evolutionary relationships. These were then named according to our phylogenetic tree (Supplementary Data Excel File 3).

**Figure 3:**
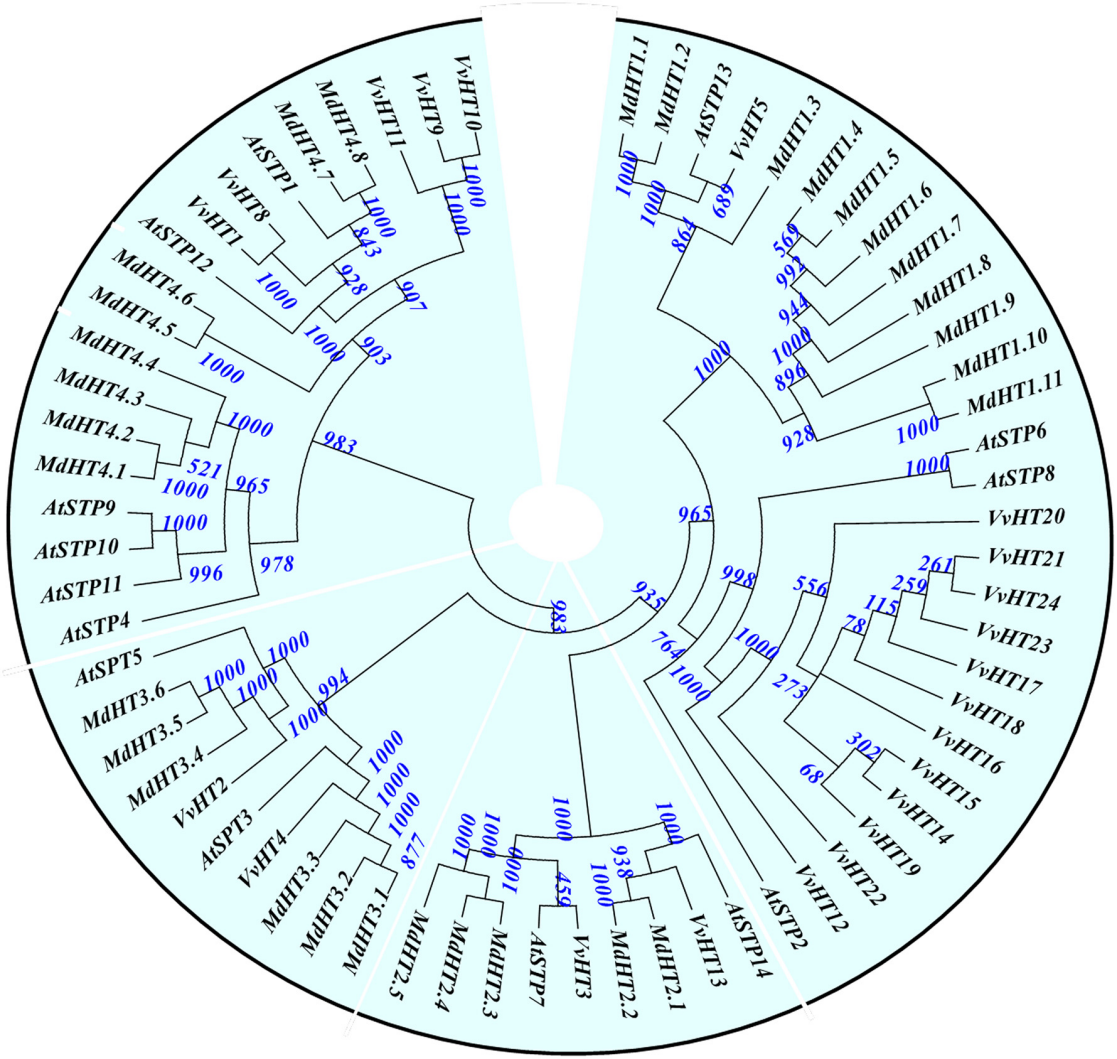
**Phylogenetic analysis of *Malus domestica MdHT* families with *Vitis vinifera* and *Arabidopsis thaliana***. Tree was constructed via Neighbor-Joining method with 1000 bootstrap replications. Accession numbers for all *Malus* genes are listed in Supplementary Data Excel File 3. Accession numbers for *Vitis* genes were reported by Afoufa-Bastien et al. (2010). Accession numbers for *A. thaliana*: AT1G11260.1/*AtSTP1*, AT1G07340.1/*AtSTP2*, AT5G61520.1/*AtSTP3*, AT3G19930.1/*AtSTP4*, AT1G34580.1/*AtSTP5*, AT3G05960.1/*AtSTP6*, AT4G02050.1/*AtSTP7*, AT5G26250.1/*AtSTP8*, AT1G50310.1/*AtSTP9*, AT3G19940.1/*AtSTP10*, AT5G23270.1/*AtSTP11*, AT4G21480.1/*AtSTP12*, AT5G26340.1/*AtSTP13*, and AT1G77210.1/*AtSTP14*.

Thirty candidate *MdHT*s were divided into four groups. The first, named the *MdHT1* subfamily, contained 11 genes (Figure 3). *MdHT1.1* through *MdHT1.3* clustered with *AtSTP13* and *VvHT5*. By contrast, *MdHT1.4*through *MdHT1.11* did not cluster with any *Arabidopsis* or *Vitis* genes. This *MdHT1* subfamily included *MdHT1.1*, which shared 95.3% similarity to the amino acid sequence of*MdHT1.2*, as well as *MdHT1.10* and −1.11, with 95.9% similarity. In the second group, five members of the *MdHT2* subfamily clustered with *AtSTP14, AtSTP7, VvHT13*, and *VvHT3*, while both *MdHT2.1* and −2.2 had higher homology with *AtSTP14* and *VvHT13*. The protein sequences of *MdHT2.3* and −2.4 differed by only two amino acids. In the third group, *MdHT3* subfamily members *MdHT3.1*through *MdHT3.3* were highly homologous with *VvHT4* and *AtSTP3*, while three other *MdHT3*s were highly conserved and clustered with *VvHT2* and *AtSTP5*. The eight *MdHT4* subfamilies that comprised the fourth group were separated into two subgroups. The first had four relatively conserved members clustered with four *AtSTP*s, based on their amino acid sequences. The second subgroup contained *MdHT4.5* and −4.6 with 89.8% similarity in a separated branch, while *MdHT4.7* and −4.8 shared 93.8% similarity with *AtSTP1* (Figure 3; Supplementary Data Excel File 3).

Within the *Malus* genome, 29 *MdHT* families were located on 10 chromosomes while *MdHT4.6*occurred on an unanchored contig. Five *MdHT1* members—*MdHT1.1*, −1.4, −1.5, −1.6, and −1.10—were in tandem on chr9 at approximately 66 kb while the other five *MdHT1*s were in tandem on chr13 at approximately 95 kb. *MdHT1.3* was located on chr7 along with *MdHT3.3*. For the other *MdHT* subfamilies, three pairs of *MdHTs* showed tandem duplications, including *MdHT2.3-MdHT2.4* on chr2, *MdHT4.1-MdHT4.2* on chr15, and *MdHT3.5-MdHT3.6* on chr13 (Supplementary Figure 1). Based on the chromosomal evolution described by Velasco et al. (2010), two pairs of very similar *MdHT* were located on homologous chromosomes, including *MdHT2.1* (chr2)-*MdHT2.2* (chr15) and *MdHT4.7* (chr5)-*MdHT4.8* (chr10) (Supplementary Figure 1).

**Figure 4:**
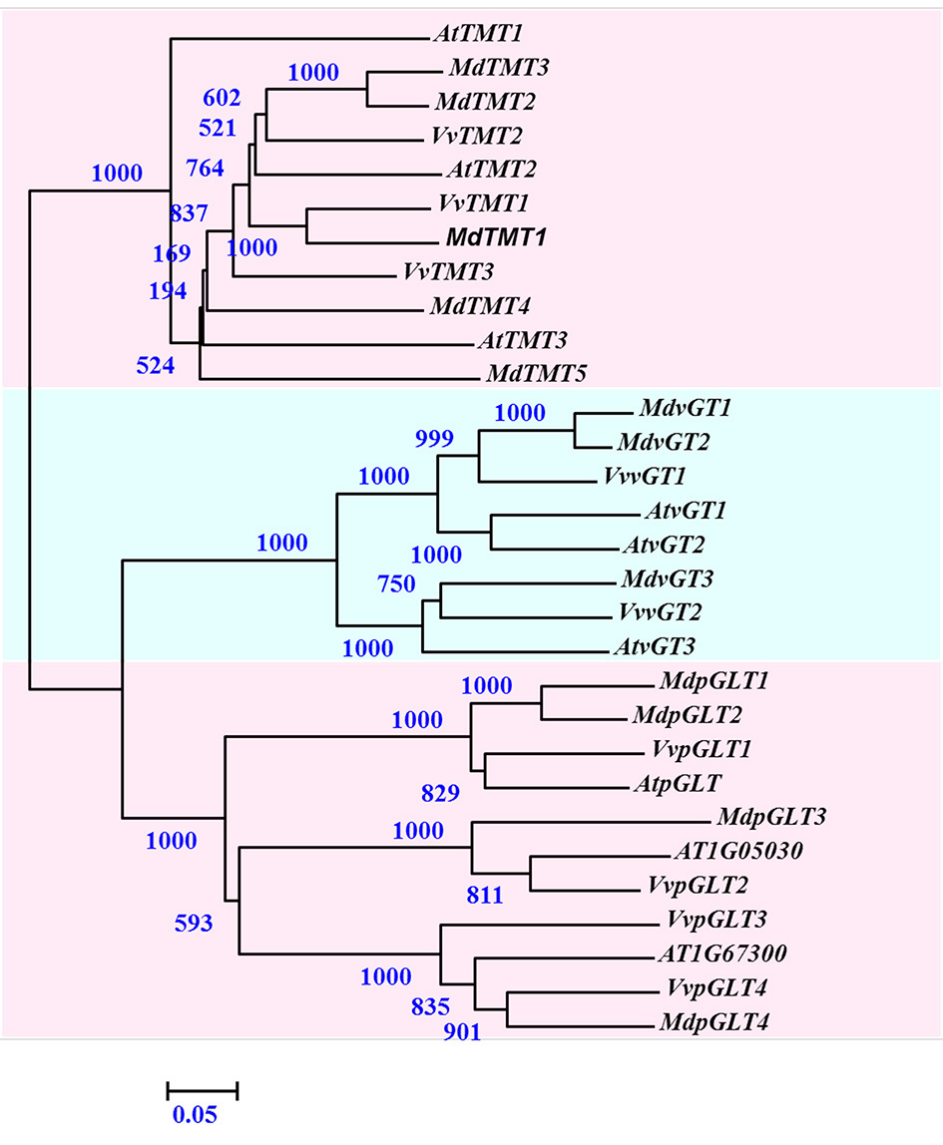
**Phylogenetic analysis of *Malus domestica MdTMT, MdvGT*, and *MdpGLT* families with *Vitis vinifera* and *Arabidopsis thaliana***. Tree was constructed via Neighbor-Joining method with 1000 bootstrap replications. Accession numbers for all *Malus* genes are listed in Supplementary Data Excel File 3. Accession numbers for *Vitis* genes were reported by Afoufa-Bastien et al. (2010). Accession numbers for *A. thaliana*: AT1G20840.1/*AtTMT1*, AT4G35300.1/*AtTMT2*, AT3G51490.2/*AtTMT3*, AT5G16150.1/*AtpGLT*, AT3G03090.1/*AtvGT1*, AT5G17010.1/*AtvGT2*, and AT5G59250.1/*AtvGT3*.

For other MST families, *MdTMT1* on chr6 had 13 ESTs in the *Malus* EST database, and showed high homology with *VvTMT1* (Figure 4). Both *MdTMT2* and −3 were 90.4% similar in their amino acid sequences, and clustered with *VvTMT2* and *AtTMT2*. The *MdTMT4* and *MdTMT5* tandems on chr9 were highly homologous to *AtTMT3*. *MdvGT1* (on chr7) was 93.2% similar to *MdvGT2* (chr1), and was orthologous to *VvvGT1* and *AtvGT1*. *MdvGT3* (chr11) was orthologous to *VvvGT2* and *AtvGT3*. Chromosome 3 had four *MdpGLT* genes, i.e., *MdpGLT1* and *MdpGLT2* with high homology to *VvpGLT1* and *AtpGLT* (At16150), as well as *MdpGLT3* (homologous to *VvpGLT2* and At05030, a putative *AtpGLT*) and *MdpGLT4* (homologous to *VvpGLT4* and At67300) (Figure 4; Supplementary Data Excel File 3).

**Figure 5:**
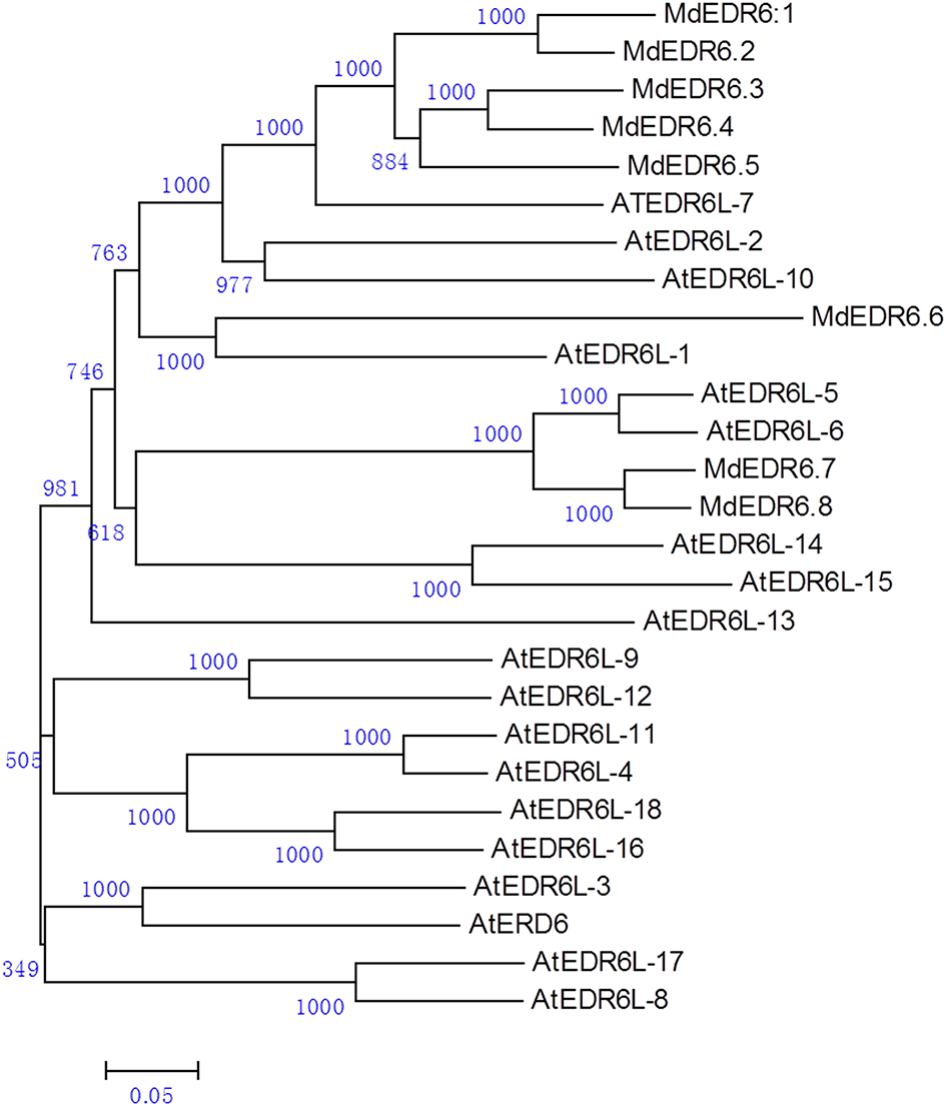
**Phylogenetic analysis of *Malus domestica MdEDR6* families with *Arabidopsis thaliana***. Tree was constructed via Neighbor-Joining method with 1000 bootstrap replications. Accession numbers for all *Malus* genes are listed in Supplementary Data Excel File 3. Accession numbers for *A. thaliana*: AT1G08930.1/*AtERD6*, AT1G54730.2/*AtEDR6L-1*, AT5G18840.1/*AtEDR6L-2*, AT1G08920.2/*AtEDR6L-3*, AT3G05160.1/*AtEDR6L-4*, AT1G19450.1/*AtEDR6L-5*, AT1G75220.1/*AtEDR6L*-*6*, AT2G48020.2/*AtEDR6L*-*7*, AT1G08900.1/*AtEDR6L*-*8*, AT3G05155.1/*AtEDR6L*-*9*, AT3G05150.1/*AtEDR6L*-*10*, AT3G05165.1/*AtEDR6L*-*11*, AT3G05400.1/*AtEDR6L*-*12*, AT3G20460.1/AtEDR6L-13, AT4G04750.1/*AtEDR6L*-*14*, AT4G04760.1/*AtEDR6L*-*15*, AT5G27350.1/*AtEDR6L*-*16*, AT1G08890.1/*AtEDR6L*-*17*, and AT5G27360.1/*AtEDR6L*-*18*.

When *AtEDR6* protein sequences were used as query, we found eight *MdEDR6* families in the *Malus* genome. Five predicted genes had highly concordant EST sequences, especially *MdEDR6.5*. Results of phylogenetic analysis with *Arabidopsis AtEDR6*-like families indicated that eight candidate *MdEDR6*s could be divided into three groups (Figure 5; Supplementary Data Excel File 3). For example, five members—*MdEDR6.1* through *MdEDR6.5*—showed high similarity and shared the same clade with *AtEDR6L-7*, whereas *MdEDR6.6* had high homology with *AtEDR6L-1*. Both *MdEDR6.7* and −6.8 were clustered with *AtEDR6L-5* and *AtEDR6L-6*. However, 14 *AtEDR6L* families plus *AtEDR6* (At1g08930) were clustered in clades that had no *Malus* genes (Figure 5). Four members—*MdEDR6.2* through *MdEDR6.5*—were in tandem at approximately 19 kb at the tail end of chr11. *MdEDR6.1* occurred on chr3, *MdEDR6.6* and −6.7 were found on chr12, and *MdEDR6*.8 was on chr8 (Supplementary Figure 1).

### *MdSWEET* genes in the *malus domestica* genome

**Figure 6:**
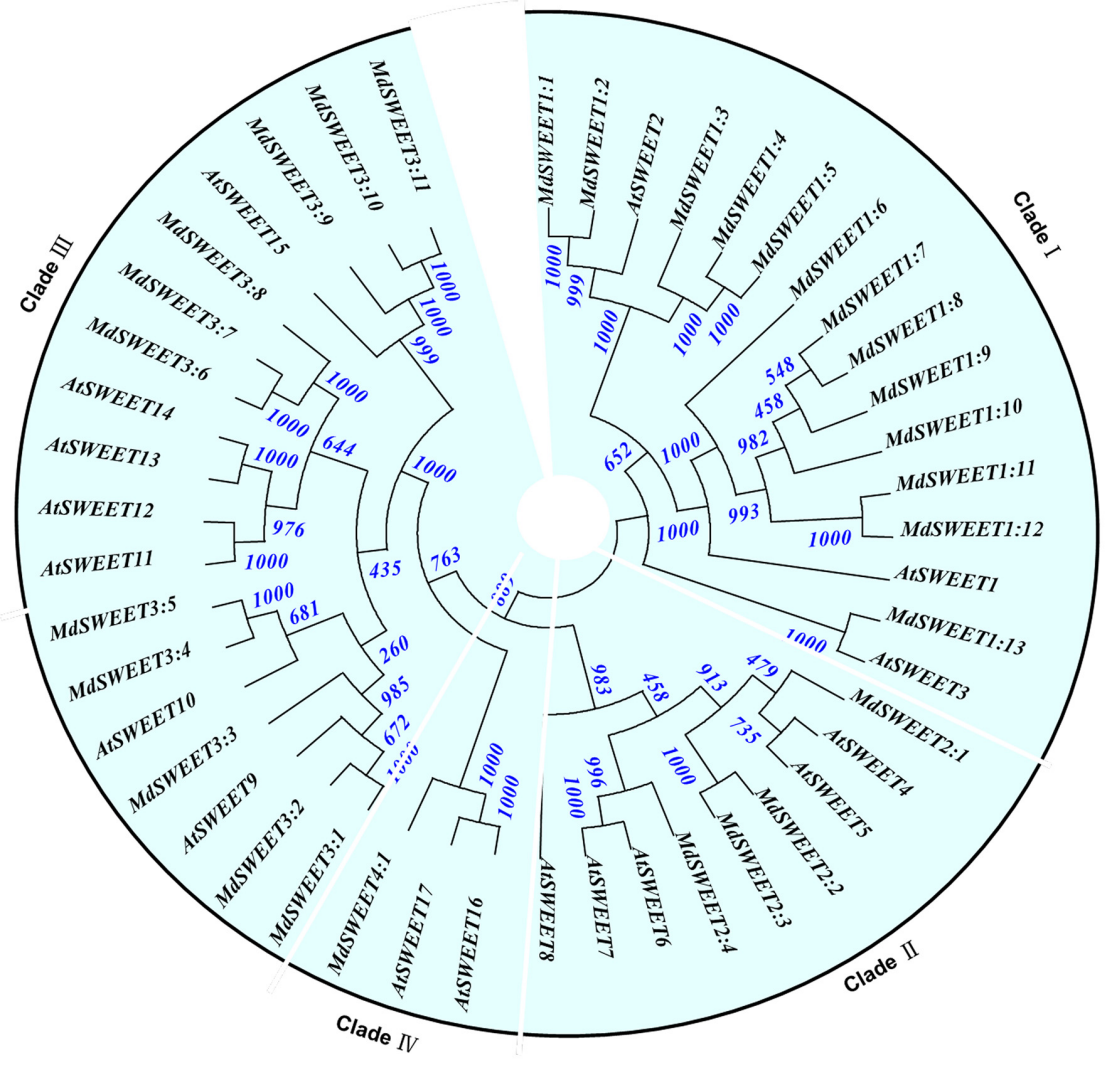
**Phylogenetic analysis of *Malus domestica MdSWEET* families with *Arabidopsis thaliana***. Tree was constructed via Neighbor-Joining method with 1000 bootstrap replications. Accession numbers for *Malus* genes are listed in Supplementary Data Excel File 4. Accession numbers for *A*. *thaliana*: AT1G21460.1/*AtSWEET1*, AT3G14770.1/*AtSWEET2*, AT5G53190.1/*AtSWEET3*, AT3G28007.1/*AtSWEET4*, AT5G6285.1/*AtSWEET5*, AT1G66770.1/*AtSWEET6*, AT4G10850.1/*AtSWEET7*, AT5G40260.1/*AtSWEET8*, AT2G39060.1/*AtSWEET9*, AT5G50790.1/*AtSWEET10*, AT3G48740.1/*AtSWEET11*, AT5G23660.1/*AtSWEET12*, AT5G50800.1/*AtSWEET13*, AT4G25010.1*/AtSWEET14*, AT5G13170.1/*AtSWEET15*, AT3G16690.1/*AtSWEET16*, and AT4G15920.1/*AtSWEET17*.

The *Malus* genome contained 33 candidate SWEET gene sets, which were revealed when 17 *AtSWEET* protein sequences were used as query. Of these, 16 had high concordant EST sequences in the *Malus* EST database. Five sets diverged in their corresponding ESTs, but concordant sequences with ESTs were found in all predictions for the *Malus* genome database (Supplementary Data Excel File 4). Another three sets had reasonably well-matched splicing sequences in all predictions. The thresholds for *MdSWEET* were set at >4 for the number of transmembrane domains and >200 a.a. for the length of the coding amino acids. Upon re-screening, 29 genes were identified as candidates, which were then aligned to elucidate their evolutionary relationships and named according to our phylogenetic tree (Figure 6; Supplementary Data Excel File 4).

Phylogenetic analysis demonstrated that the 29 families could be separated into four groups: *MdSWEET1, MdSWEET2, MdSWEET3*, and *MdSWEET4* (Figure 6), similar to what has been reported previously with *Arabidopsis* and rice (Chen et al., 2010). The *MdSWEET1* subfamily contained 13 members. Of these, *MdSWEET1.1* through *MdSWEET1.5* were clustered in a clade with *AtSWEET2*. The amino acid sequence of *MdSWEET1.2* had 43 more residues of amino acid at 5′ end compared with *MdSWEET1.1*. *MdSWEET1.4* and −1.5 were 93.1% similar. *MdSWEET6* through *MdSWEET12* were homologous to *AtSWEET1*, and had >83.5% similarity among their amino acid sequences. As a single, *AtSWEET1.13* clustered with *AtSWEET3*. Of the four candidates in the *MdSWEET2* subfamily, *MdSWEET2.1* showed high homology with *AtSWEET4* and −5, but was also closely homologous to *MdSWEET2.2* and −2.3. *MdSWEET2.4* showed high homology with *AtSWEET7* and −8. In the *MdSWEET3* subfamily, *MdSWEET3.1* and −3.2 (87.3% similarity) were clustered with *AtSWEET9* and *MdSWEET3.3*. *MdSWEET3.4* and −3.6 (88.1% similarity) were homologous to *AtSWEET10*. Both *MdSWEET3.6* and −3.7 were 88.3% similar to *MdSWEET3.8*, and clustered with *AtSWEET11* through *AtSWEET14*. *MdSWEET3.9* clustered with *AtSWEET15* and showed 85.8% similarity with *MdSWEET3.10* and −3.11, both of which had the same sequence. In the fourth subfamily, *MdSWEET4.1* was grouped with *AtSWEET16* and −17 (Figure 6; Supplementary Data Excel File 4).

In all, these 29 *MdSWEET* genes were distributed on 15 chromosomes plus one anchored contig. Four pairs of *MdHT* arose from tandem duplications, including *MdSWEET1.1-MdSWEET1.2* on chr3, *MdSWEET1.9-MdSWEET1.10* on chr7, *MdSWEET3.7-MdSWEET3.8* on chr14, and *MdSWEET3.11-MdSWEET3.12* on chr16 (Supplementary Figure 1).

### Expression profiling of sugar transporters in different tissues of apple

**Figure 7:**
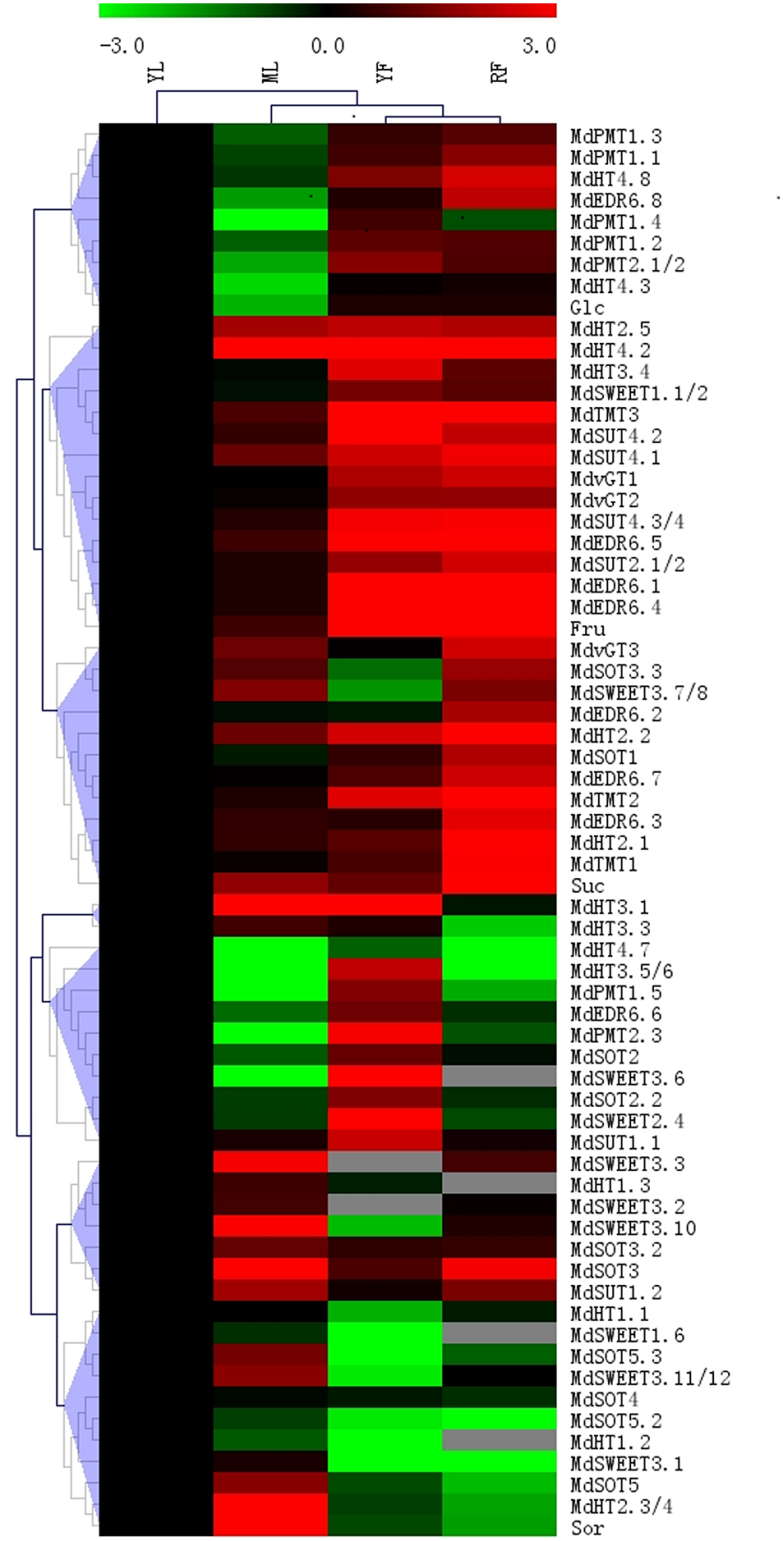
**Heat map illustrating sugar concentrations and transporter expression levels in shoot tips, mature leaves, young fruit (16 DAB), and mature fruit (122 DAB) from “Gala” apple**. Relative expression abundances and sugar concentration are shown in Supplementary Data Excel Files 6, 7. Fold-difference is designated as log2 value, while the data in shoot tip was set as “1” for each gene and sugar. Genes with similar profiles across arrays are grouped on top by hierarchical-clustering method.

To determine the tissue-specific expression levels of candidate genes, we performed qRT-PCR to analyze mRNA relative abundance in mature leaves, shoot tips, young fruit (16 DAB), and completely ripened fruit (122 DAB) from “Gala” apple (Supplementary Data Excel File 6). Transcripts of 10 *MdSOT* and seven *MdPMT* subfamilies were examined. Expression of *MdSOT3, MdSOT5*, and *MdSOT5.3* was strongest in mature leaves, whereas transcripts for *MdSOT1, MdSOT3.3*, and *MdPMT1.1* were most abundant in ripened fruit and those of *MdSOT2* and *MdPMTs* were at their highest levels in young fruit (Figure 7).

Expression was detected for 11 members of the *MdHT* family. However, *MdHT1.2* and −1.3 were barely detected in the fruit, especially those that were already ripened. *MdHT2*.*1*, −2.2, and −4.8 showed the highest abundance in older fruit, while *MdHT3.4* and *MdHT3.5/6* were most strongly detected in young fruit, *MdHT2.3/4* in mature leaves, and *MdHT1.2* and *MdHT4.7* in young leaves (Figure 7; Supplementary Data Excel File 6).

Except for *MdEDR6.6* (most abundant in young fruit), the seven *MdEDR* family members showed their highest levels of expression in ripened fruit. For those genes, expression was not distinctly different between young and mature leaves. Transcripts of*MdTMT1, MdTMT2*, and *MdvGT3* were most abundant in ripened fruit while those of *MdTMT3* were highest in young fruit (Supplementary Data Excel File 6).

Using 10 pairs of primers, we detected a high level of expression by various *MdSWEETs* in mature leaves. Expression by *MdSWEET1*.6 and −3.5 was not abundant in ripened fruit or by *MdSWEET3.1* and −3.2 in young fruit. Transcript levels were much higher for *MdSWEET1.1/2, MdSWEET2.4*, and *MdSWEET3.5* in young fruit and for *MdSWEET3.1* and *MdSWEET3*.*10/11* in mature leaves (Supplementary Data Excel File 6).

Except for *MdSUT1.2* in mature leaves, expression of *MdSUT*s was higher in fruit tissues. In particular, *MdSUT1.1* and *MdSUT4.2* transcripts were more abundant in the young fruit while those of *MdSUT2.1/2* and *MdSUT4.1* were at higher levels in ripened fruit (Supplementary Data Excel File 6).

When we investigated possible correlations between gene expression and sugars (Figure 7), we found that transcripts of *MdSOT* members, especially *MdSOT5*, paralleled that of Sor transport and accumulations. The expression of most *MdEDR6* members (except *MdEDR6.6* and −6.8) showed parallel trends for Suc and Fru concentrations, particularly in the fruit. Other genes, including *MdTMT1, MdTMT2, MdHT2.1*, and *MdHT2.2*, were closely associated with Fru and Suc concentrations.

### The relationship between expression abundance and the concentration of main sugars during fruit development

**Figure 8:**
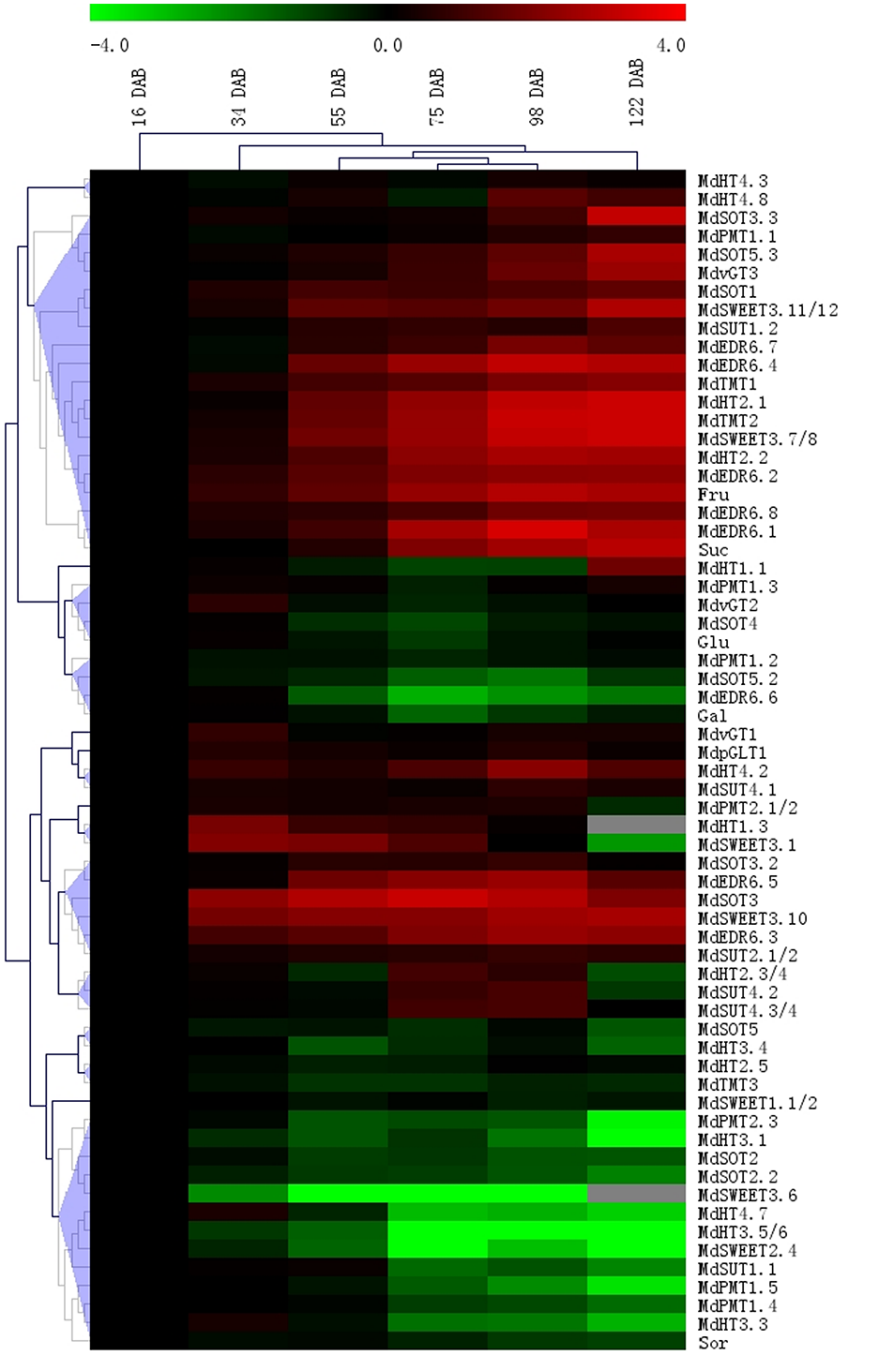
**Heat map illustrating sugar concentrations and transporter expression levels in fruits of “Gala” apple at different developmental stages**. Relative expression abundances, measured with qRT-PCR, are shown in Supplementary Data Excel Files 6, 7. Fold-difference is designated as log2 value, while the data in young fruit (16 DAB) s was set as “1” for each gene and sugar. Genes with similar profiles across arrays are grouped on top by hierarchical-clustering method.

To investigate the relationship between gene expression and sugar accumulations, we assayed fruits at different stages of development (Figure 8; Supplementary Data Excel Files 6, 7). Overall, five general patterns emerged. In the first, and largest, group, expression by 17 genes increased in parallel with higher concentrations of Fru and Suc. This was especially true for *MdTMT1, MdTMT2, MdHT2.1, MdHT2.2, MdSWEET3.6/7*, and five *MdEDR*s, for which expression was significantly and positively correlated with levels of Suc and Fru in ripened fruit (Table 1). Concentrations of Sor decreased over time in conjunction with diminished expression by 12 genes, such as *MdHT3.3, MdPMT1.4, MdPMT1.5, MdSUT1.1, MdSWEET2.4, MdSOT2*, and *MdSOT5*. In addition, Glc concentrations were significantly and positively correlated with *MdvGT2* and *MdSOT4* in developed fruit, while Gal concentrations were correlated with the expression of *MdEDR6.6* and *MdSOT5.2* (Table 1; Figure 8).

**Table 1:** **Correlation coefficients between expression abundance and main sugars or sugar alcohols during development of apple fruit (*n* = 6)**.

	**Glc**	**Fru**	**Suc**	**Gal**	**Sor**		**Glc**	**Fru**	**Suc**	**Gal**	**Sor**
*MdHT1.1*	0.439	0.241	0.558	0.234	−0.446	*MdSWEET1.1/2*	0.249	−0.790	−0.707	0.406	0.692
*MdHT1.2*	0.407	−0.842^*^	−0.866^*^	0.737	0.869^*^	*MdSWEET1.6*	0.709	−0.809	−0.688	0.768	0.682
*MdHT1.3*	0.177	−0.538	−0.691	0.305	0.528	*MdSWEET2.4*	0.622	−0.897^*^	−0.795	0.801	0.843^*^
*MdHT2.1*	−0.274	0.958^**^	0.987^**^	−0.587	−0.971^**^	*MdSWEET4.1*	−0.004	−0.525	−0.705	0.242	0.572
*MdHT2.2*	−0.452	0.997^**^	0.948^**^	−0.753	−0.968^**^	*MdSWEET3.1*	−0.017	0.728	0.844^*^	−0.234	−0.774
*MdHT2.3/4*	−0.651	0.245	0.012	−0.696	−0.133	*MdSWEET3.2*	0.074	0.785	0.945^**^	−0.269	−0.888^*^
*MdHT2.5*	0.636	0.003	0.141	0.385	−0.106	*MdSWEET3.5*	0.445	−0.706	−0.553	0.578	0.665
*MdHT4.2*	−0.262	0.850^*^	0.689	−0.581	−0.790	*MdSWEET3.6/7*	−0.307	0.968^**^	0.977^**^	−0.609	−0.967^**^
*MdHT4.3*	−0.134	0.612	0.515	−0.242	−0.485	*MdSWEET3.9*	−0.261	0.869^*^	0.812^*^	−0.517	−0.876^*^
*MdHT4.7*	0.666	−0.907^*^	−0.860^*^	0.844^*^	0.829^*^	*MdSWEET3.1011*	−0.058	0.741	0.888^*^	−0.301	−0.820^*^
*MdHT4.8*	0.091	0.749	0.730	−0.202	−0.730	*MdSUT1.1*	0.457	−0.885^*^	−0.920^**^	0.763	0.911^*^
*MdHT3.1*	0.135	−0.808	−0.810	0.371	0.833^*^	*MdSUT1.2*	−0.353	0.761	0.857^*^	−0.524	−0.791
*MdHT3.3*	0.569	−0.923^**^	−0.919^**^	0.798	0.883^*^	*MdSUT2.1/2*	−0.472	0.954^**^	0.820^*^	−0.716	−0.891^*^
*MdHT3.4*	0.383	−0.445	−0.496	0.341	0.420	*MdSUT4.1*	0.032	0.723	0.592	−0.263	−0.698
*MdHT3.5/6*	0.565	−0.915^*^	−0.802	0.785	0.875^*^	*MdSUT4.2*	−0.597	0.455	0.169	−0.700	−0.296
*MdEDR6.1*	−0.449	0.962^**^	0.849^*^	−0.756	−0.899^*^	*MdSUT4.3/4*	−0.687	0.668	0.433	−0.858^*^	−0.539
*MdEDR6.2*	−0.522	0.981^**^	0.925^**^	−0.784	−0.949^**^	*MdSOT1*	−0.271	0.856^*^	0.866^*^	−0.488	−0.861^*^
*MdEDR6.3*	−0.455	0.993^**^	0.905^*^	−0.753	−0.960^**^	*MdSOT2*	0.484	−0.896^*^	−0.808	0.653	0.828^*^
*MdEDR6.4*	−0.463	0.993^**^	0.921^**^	−0.742	−0.937^**^	*MdSOT2.2*	0.335	−0.879^*^	−0.848^*^	0.566	0.882^*^
*MdEDR6.5*	−0.781	0.818^*^	0.567	−0.884^*^	−0.640	*MdSOT3*	−0.806	0.534	0.245	−0.793	−0.380
*MdEDR6.8*	−0.200	0.961^**^	0.972^**^	−0.546	−0.984^**^	*MdSOT3.2*	−0.718	0.571	0.241	−0.71	−0.345
*MdEDR6.7*	−0.314	0.953^**^	0.877^*^	−0.610	−0.895^*^	*MdSOT3.3*	0.233	0.528	0.784	−0.033	−0.691
*MdEDR6.6*	0.772	−0.857^*^	−0.725	0.865^*^	0.732	*MdSOT4*	0.959^**^	−0.500	−0.292	0.849^*^	0.314
*MdTMT1*	−0.257	0.951^**^	0.972^**^	−0.560	−0.964^**^	*MdSOT5*	0.168	−0.475	−0.631	0.329	0.607
*MdTMT2*	−0.300	0.976^**^	0.974^**^	−0.616	−0.971^**^	*MdSOT5.2*	0.688	−0.907^*^	−0.709	0.895^*^	0.805
*MdTMT3*	0.691	−0.635	−0.484	0.693	0.537	*MdSOT5.3*	0.075	0.698	0.897^*^	−0.219	−0.822^*^
*MdvGT1*	0.569	0.025	0.064	0.304	−0.200	*MdPMT1.1*	−0.059	0.849^*^	0.974^**^	−0.390	−0.915^*^
*MdvGT2*	0.837^*^	−0.503	−0.379	0.760	0.311	*MdPMT1.2*	0.734	−0.455	−0.205	0.770	0.362
*MdvGT3*	0.001	0.793	0.951^**^	−0.318	−0.889^*^	*MdPMT2.1/2*	−0.361	−0.092	−0.431	−0.259	0.251
*MdpGLT1*	0.165	0.233	0.025	0.015	−0.213	*MdPMT2.3*	0.427	−0.837^*^	−0.837^*^	0.583	0.810
*MdpGLT2*	−0.358	0.764	0.531	−0.559	−0.625	*MdPMT1.3*	0.786	0.050	0.239	0.612	−0.202
*MdpGLT3*	0.319	0.678	0.775	−0.067	−0.765	*MdPMT1.4*	0.426	−0.947^**^	−0.974^**^	0.729	0.960^**^
*MdpGLT4*	−0.558	0.939^**^	0.776	−0.781	−0.820^*^	*MdPMT1.5*	0.439	−0.969^**^	−0.974^**^	0.727	0.963^**^

* Significant at P ≤ 0.05;

** *Significant at P ≤ 0.01*.

## Discussion

Domesticated apple genotypes are very heterozygous, making genome sequencing difficult (Velasco et al., 2010). As a result, predicted genes in the *Malus* genome database contain redundant data. Using protein sequences of *AtPMT* and reported *MdSOT* (Gao et al., 2005; Fan et al., 2009) as query, we obtained 40 consensus putative SOT gene sets from the *Malus* database, which is more than found for apple by Velasco et al. (2010) and much more than those described for peach (Verde et al., 2013). As thresholds, we used *Malus* ESTs, and set sequence size and number of transmembrane domains as high as possible. Putative gene sets of insufficient size or with too few transmembrane domains did not allow one to find ESTs in the GenBank database. After screening, 27 of 40 putative SOT genes were chosen as possible members of the *MdSOT* and *MdPMT* families. The same selections were done for *MST*s, *SUT*s, and *SWEET*s. Although it is not likely that these genes are the only ones that exist, we are confident that they are functional because we found all of them to be expressed in *Malus*, and that they represent most of the genes expressed in apple leaves, shoot tips, and fruit.

### *Rosaceae* plants have specific sorbitol transporters

In *Rosaceae*, photosynthesis-derived carbohydrates are transported mainly as Sor, which is mediated by SOT, a subfamily of the *PMT* family (Fan et al., 2009). Compared with other plant genomes, apple has considerably more copies of key genes related to Sor metabolism, including *aldose 6-P reductase* (*A6PR*), *sorbitol-dehydrogenase* (*SDH*), and *SOT* (Velasco et al., 2010). Both SDH and SOT, which are highly specific to *Rosaceae* fruits, have evolutionarily been shown to be large families of specific paralogous genes. After clustering to elucidate the evolutionary relationship with *AtPMT* and *VvPMT* families, we were able to divide the 27 candidate genes into two groups: *MdSOTs* and *MdPMTs*, and *MdSOTs* group contained all reported functional *MdSOT*s but lacked any *PMT* from *Arabidopsis* and *Vitis*. Our 10 *MdSOT*s (including highly expressed *MdSOT2*, −4, and −5; Gao et al., 2005) were in tandem at approximately 449 kb on chr12. However, no *MdSOT*s or *MdPMT*s were found on chr4 and chr14, both of which share partial homology with large segments on chr12 (Velasco et al., 2010). Reported members of *MdSOT* families have greater substrate selectivity for Sor (Watari et al., 2004; Gao et al., 2005). All of our selected *MdPMT* members had lower transcript abundance in mature leaves that contained a high Sor concentration. These results implied that the segment on chr12 is related evolutionarily to Sor metabolism, and that a trend toward Sor accumulation may have been somewhat based on gene duplication that created large families of *MdSOT* paralogous genes. Many *PMT* subfamily members in *Arabidopsis* transport a wide range of sugar alcohols and hexoses, and may be non-specific for sorbitol (Klepek et al., 2010). Supporting this, we determined that the expression of *MdPMT1.1* and *-1.2* was highly correlated with Fru and Suc concentrations in developed fruit, and that expression of seven *MdPMT*s was relatively higher in fruits than in leaves. Further confirmation is needed for the function of *MdPMT* in conferring substrate selectivity for Sor or other sugar alcohols and hexoses.

### Carbohydrate unloading in apple is driven by SOT, SUT, and HT families

After their synthesis in apple leaves, both Sor and Suc are passively loaded into SE-CC of the phloem via the symplasm pathway (Reidel et al., 2009), and then unloaded in the fruit to the parenchyma cells through the apoplasm pathway (Zhang et al., 2004). Therefore, both SOT and SUT may not be essential for phloem loading, but serve as proton co-transporters for phloem unloading. This theory is supported by reports of higher transcript abundances for *MdSOT1* and −2 (Gao et al., 2005) and our finding of a *MdSUT4* clade and four *MdPMT1* members in fruit. Although *AtSUT4*, as an antiporter, is located on the tonoplast (Schneider et al., 2011), *MdSUT4.1* is present on the plasma membrane and functions in unloading Suc to the apoplasm (Fan et al., 2009). Many *MdSOT*s, e.g., *MdSOT3*, −4, −5, and −5.3, are expressed in the vascular tissue of source leaves (Watari et al., 2004). They might not play direct roles in the movement of sugar alcohols or phloem loading. When highly expressed in the leaves, *MdSOT* and *MdSUT* may be involved in recovering leaked Suc or Sor, local transport via the apoplasm, or regulation of osmotic potential and turgor among intracellular compartments by adjusting concentrations to adapt to changes in environmental conditions, such as drought (Li et al., 2012).

Most of the *HT*s or *STP*s identified in *Arabidopsis* are localized to the plasma membrane and are capable of transporting hexoses, such as Glc, Fru, and Gal. In apple fruit, after Suc is unloaded, it can be hydrolyzed to Glc and Fru by cell wall invertase. *MdHT* is needed for moving Glc and Fru from cell wall spaces into parenchyma cells because Sor transport is inhibited by high concentrations of hexoses (Gao et al., 2003). We detected mRNA expression of *MdHT2.1* and −2.2 were more abundant in the fruit and were significantly correlated with Fru and Suc concentrations. This is the same trend noted for *VvHT13*, a highly orthologous gene in grape (Afoufa-Bastien et al., 2010). However, their homologous gene in *Arabidopsis* is *AtSTP14*, which is galactose substrate-specific (Poschet et al., 2010). Expression of *MdHT4.4* was also highly correlated with Fru concentrations in developed fruit. This gene has high homology with *AtSTP9*, which is Glc substrate-specific (Schneidereit et al., 2003). These results implied that the functions of these three *MdHT*s are different in apple when compared with their orthologs in *Arabidopsis*. *MdHT4.8* was highly expressed in ripened fruit and showed high homology to *AtSTP1, VvHT1*, and *VvHT8. AtSTP1* transports Glc as well as Gal, mannose, and xylose (Büttner, 2010). *AtSTP1*, −13, and −14 function in the recovery of hexoses generated during the process of cell wall remodeling and because of passive leakage by sugars from cells (Yamada et al., 2011). Stronger expression of *MdHT2.1*, −2.2, and −4.8 in ripened apple fruit may be related to the process of hexose release (including that of Gal, xylose, and Glc) due to cell wall degradation. In young fruit, transcript levels for *MdHT3.5/6* and *MdHT3.4* were high, and those genes showed the closest similarity to *AtSTP5* (uncharacterized) in *Arabidopsis* and *VvHT2* in *Vitis*. Although *VvHT2* is weakly expressed in grape leaves, transcript levels are high in young berries but decline around veraison (Afoufa-Bastien et al., 2010). That pattern resembles what we observed for *MdHT3.5/6* expression in apple. In tomato fruit, three HT genes are co-localized with QTLs for sugar accumulation (Prudent et al., 2011). That relationship has been further verified by RNAi knockdowns of three *LeHT*s (McCurdy et al., 2010) that are highly homologous to *MdHT4.8, MdHT2.1/2.2*, and *MdHT1.1*. *AtSTP13*, highly homologous to *MdHT1.1/2*, can transport Glc, Fru, and Gal in yeast (Yamada et al., 2011). Here, *MdHT1.1* expression was strongly up-regulated in mature fruit whereas *MdHT1.2* was barely detectable in any fruit. Future studies should focus on the roles that *MdHT*s have in sugar accumulation and efficient unloading of carbohydrates.

### Expression of *MdvGT*s, *MdTMt*s, *MdEDR6*, and *MdSWEET*s is correlated with sugar accumulations in apple fruit vacuoles

For apple, most of the Fru, Suc, and Glc are stored in the central vacuoles of parenchyma cells (Yamaki and Ino, 1992). This requires that sugars be transported from the cytosol by carrier proteins localized on the tonoplast membrane. In *Arabidopsis*, these vacuolar proteins are encoded by *AtvGT* (Aluri and Büttner, 2007), *AtTMT* (Wormit et al., 2006), *AtEDR6* (Poschet et al., 2011), and *AtSWEET17* (Chardon et al., 2013). We found three *MdvGT*s with high homology to three *AtvGT*s; their expression was much stronger in fruit than in leaves. We have previously reported (Li et al., 2012) that *MdvGT1* and *MdvGT2* transcript levels are highest in young fruit and are correlated with Glc concentrations in ripened fruit. *MdvGT3*, which presents an N-terminal extension as *VvvGT2* (Afoufa-Bastien et al., 2010), is more closely related to *AtvGT3* and *VvvGT2*. Its expression is highly correlated with Suc concentrations in fruit. By contrast, expression of *MdvGT3* was stronger in mature leaves where less sugar was accumulated. Its predicted locations were primarily the chloroplasts and mitochondrial membrane. Although *AtvGT1* mediates low-affinity Fru uptake when expressed in yeast vacuoles (Aluri and Büttner, 2007), we found no correlation between *MdvGT1/2* expression and Fru uptake in apple vacuoles. These results suggested that *MdvGT* is not active in the accumulation of Fru and Suc within ripened fruit but may be involved in Glc transport.

*Arabidopsis AtTMT1*and *AtTMT2* are equally capable of moving Glc and Suc into the vacuoles (Schulz et al., 2011). The former is associated with a rate of Fru transport that is approximately 30% of that for Glc in yeast (Wormit et al., 2006). Two of three grape TMTs—*VvTMT1* and −2—are strongly expressed in post-veraison berries, a period that coincides with the major phase of hexose accumulation (Afoufa-Bastien et al., 2010). We previously reported that, although 18 predicted genes in apple genome are similar to *AtTMT*s, we were able to select only five *MdTMTs* based on ESTs, length of mRNA, and number of transmembrane domains. Transcript levels of *MdTMT1* and −2, both of which share high similarity in amino acids with *AtTMT1/2* and *VvTMT1/2*, paralleled Fru and Suc concentration, suggesting that both proteins are involved in transporting those sugars into the vacuoles. Therefore, they may be highly efficient Fru-specific vacuolar transporters in apple. This should be clarified in future examinations.

*AtERD6* and *AtESL1* (or *AtEDR6L-3*) are vacuolar exporters of Glc (Yamada et al., 2010; Poschet et al., 2011; Klemens et al., 2014). We found 16 predicted genes in the *Malus* genome with homology to *AtEDR6*. However, only eight had amino acids or trans-membrane domains that were of similar size or number. Expression by the seven selected *MdEDR* families was highest in ripened fruit, and was strongly and positively correlated with Fru and Suc concentrations but negatively with Glc concentrations. By contrast, *MdEDR6.6* was highly expressed in young fruit. In pineapple, *AcMST1* (EF460876 in GenBank) has high homology with *MdEDR6.7* and −6.8 and is located on the tonoplast; its expression is highest in fruit tissues (Antony et al., 2008). If the substrate for *MdEDR* is specific to Glc, we expect that a lower Glc concentration in fruit vacuoles would be related to greater *MdEDR6* expression in ripened fruit. Similar to *AtTMT* (Schulz et al., 2011), the protein encoded by *MdTMT* may be capable of transporting Glc and Suc into the vacuoles. Consequently, Glc moves into the vacuoles of ripened fruit along with Fru and Suc. However, because fruit sweetness has been artificially, rather than evolutionarily or naturally, selected, *EDR6* may be capable of moving Glc from the vacuoles to maintain turgor and permit a greater accumulation of Fru, which is the sweetest sugar. This phenomenon has also been suggested for pineapple, where the high amount of *EDR6* transcript in the leaves accommodates the efflux of Glc and Fru at night to provide substrates for dark-uptake of CO_2_ (Antony et al., 2008). Further characterization of *MdTMT* functioning would resolve this question.

The recently identified SWEET superfamily has 17 members in *Arabidopsis* and 21 in rice (Chen et al., 2010; Xuan et al., 2013). Here, we selected 29 genes as candidates in the *Malus* genome. Although the number of *MdSWEET* members related to special carbohydrate metabolism and higher Sor concentrations may in fact be larger in *Malus*, we detected the transcripts of only 10 *MdSWEET*s in apple leaves and/or fruit. Plant SWEET proteins are grouped into four subclades, based on amino acid homologies (Chen et al., 2010). Compared with the three *AtSWEET*s in *Arabidopsis*, 13 *MdSWEET1* subfamilies are clustered in Clade I, in which SWEET proteins mainly transport monosaccharides (Chen et al., 2010). Expression of *MdSWEET1.1/2* was twice as high in the fruit when compared with the leaves and shoot tips, which suggested that it may not be affected by Glc, Fru, and Suc concentrations in apple cells. Therefore, we hypothesized that *MdSWEET1.1/2* encodes the only *MdSWEET* protein in apple for transporting Sor, serving as a uniporter with both uptake and efflux activities. Compared with *MdSWEET1.1*, more than 45 residues at the 5′-end possibly target *MdSWEET1.2* to the tonoplasts, based on our predicted localization.

For Clade II, only *MdSWEET2.4* was detected, especially in young fruit. Its *Arabidopsis* homolog *ATSWEET8* is essential for pollen viability and is co-opted by pathogens, which likely provide an energy source and carbon at the site of infection by mediating Glc transport (Chen et al., 2010). Another gene, *OsSWEET5* in rice, is located on the plasma membrane and is homologous to *AtSWEET4*, which encodes a Gal transporter in yeast. Those earlier observations support our finding that *MdSWEET2*.*4* expression was correlated with Glc and Gal concentrations during fruit development.

In Clade III, *SWEET*s in *Arabidopsis* and rice transport disaccharides, mainly Suc (Chen et al., 2010, 2012). In particular, *AtSWEET11* and *-12* mediate the key step of Suc efflux from phloem parenchyma cells for translocation via the apoplast loading pathway (Chen et al., 2012). *AtSWEET9* is essential for nectar production and can function as a Suc efflux transporter (Lin et al., 2014). However, in apple leaves, loadings of Suc and Sor occur through the symplast pathway, which does not require a SWEET gene to mediate Suc efflux from the parenchyma to extracellular spaces. In our *MdSWEET3* clade, *MdSWEET3.6*, −3.7, and −3.8 were most similar (48%) to *AtSWEET11* and *12*. Expression of *MdSWEET3.6/7* was higher in mature leaves and ripened fruits. Similar trends were observed for transcripts of *MdSWEET3.9* and −3.10/11, which are homologous to *AtSWEET15*, a gene strongly induced by leaf senescence (Quirino et al., 1999). As homologs of *AtSWEET9, MdSWEET3.1* and −3.2 showed the highest abundance in developed fruits whereas *MdSWEET3.5* (homologous to *AtSWEET10*) had the highest expression in tissues undergoing rapid growth, such as shoot tips and young fruit. Different expression models have linked their functions with the maintenance of a balance in Suc efflux within various tissues and under certain conditions. In apple fruit, Suc and Sor unloading is accomplished through the apoplast pathway (Zhang et al., 2004), which requires one transporter to move Suc from SE-CC into the extracellular spaces. The *MdSWEET*s of Clade III, particularly *MdSWEET3.5*, may be the most likely candidate for this process.

Two members of Clade IV—*SWEET16* and −17—are localized to the tonoplast, where they have key roles in exporting Fru from the vacuoles of *Arabidopsis* leaves (Chardon et al., 2013; Klemens et al., 2013). *MdSWEET4.1* was most highly expressed in the leaves, where Fru accumulations were lower, while transcripts were reduced in the fruit, where Fru accumulation was high. This decrease in *MdSWEET4.1* expression might explain why apple fruit would have elevated Fru concentrations, as has also been demonstrated with an *Arabidopsis* mutant of *AtSWEET17* that accumulates Fru in its leaf vacuoles (Chardon et al., 2013).

### Genes function to transport sugars in apple leaves

In mature leaves, transporters are necessary in both the plastid membranes and the tonoplasts. Plasma-membrane-localized sugar transporters (e.g., *MdHT*s, *MdSUT, MdSWEET*s) assist in exporting or importing sugars to control the osmotic balance and sugar signaling between extra- and intra-cellular compartments (Linka and Weber, 2010). In apple leaves, high expression of *MdSOT3* and −5 may serve to retrieve Sor that is passively leaked into the apoplast space in source cells, or to transport Sor into the guard cells. This can help modulate stomatal apertures during periods of dehydration stress. Under drought conditions, *MdSOT*s are up-regulated in apple leaves (Li et al., 2011). Additionally, *AtSTP1*, −4, −5, and −13 are localized to the guard cells (Nørholm et al., 2006; Büttner, 2010). We detected *MdHT*s in our mature apple leaves, with expression being strongest for*MdHT2.3/4*. Future examinations will focus on their roles in mature leaves, especially under stress conditions.

Tonoplast transporters help control osmotic balance and sugar homeostasis in the cytosol by exporting or importing soluble carbohydrates in the vacuoles (Wingenter et al., 2010). We might associate this phenomenon with the high levels of expression by *MdvGT*s, *MdTMT1*, and *MdSWEET4*.*1* in our mature apple leaves. In *Arabidopsis*, the study of tmt1-2::tDNA double mutants suggests that *AtTMT1* and −2 have roles in both cellular carbon balance and whole-plant carbohydrate partitioning (Wingenter et al., 2010). Some members of the *vGT* subfamily may have functions that resemble those of the *TMT*s, as evidenced by their expression in similar tissues and their preference for transporting glucose and, to a lesser extent, fructose (Aluri and Büttner, 2007). *Arabidopsis* plants over-expressing *EDR6* from pineapple accumulate fewer monosaccharides and become sensitive to low-temperature stress (Klemens et al., 2014). These reports suggest that *EDR6* functions in osmotic adjustments by regulating sugar flux within cells. Similar results have been found with *AtSWEET17* and *AtSWEET16* (Chardon et al., 2013; Klemens et al., 2013). Therefore, we might speculate that altering the expression of *MdTMT, MdvGT, MdEDR6*, and *MdSWEET4.1* could affect plant growth and tolerance to abiotic stresses. Future experiments could involve using these genes to regulate the sugar concentration of fruits from transgenic plants in which a special promoter has been utilized in the transformation procedure.

In conclusion, this study represents a comprehensive investigation of sugar transporter genes in a woody plant. Our gene identifications and comparative analysis with transporters from *Arabidopsis* and *Vitis* indicate their strong conservation between herbaceous and woody species, as well as the expansion of particular functional subfamilies, e.g., *MdSOT, MdSWEET1*, and *MdTMT* transporters. After investigating their expression profiles simultaneously in various tissue types, and analyzing any correlations between transcript levels and the amounts of Fru and Suc in the fruit, we propose that the high accumulation of Fru is a result of coordination and cooperation by *MdTMT1*/*2* and *MdEDR6* with reduced *MdSWEET4.1* expression and greater Sor-associated Fru flux. In addition, we suggest that *MdSOT1, MdSOT2*, and *MdSUT4* are involved in the efficient unloading of Sor and Suc in fruit. Furthermore, other transporters may be required for unloading Suc and Sor into the cell wall spaces (possibly *MdSWEET1*s or *MdSWEET3*s), loading hexose from the cell wall spaces into the cytosol for storage (*MdHT*s), retrieving sugars that passively leak into the apoplast space, fine-tuning sugar flux for homoeostasis, and regulating stomatal apertures. Our current findings serve as tools for elucidating the biological function of transporters in *Malus domestica*, particularly during fruit formation and sugar accumulation. This information will have a significant impact on our knowledge of sugar transporters in general, and the development of sweetness properties in particular. However, the possible errors in the apple genome sequence may also affect the location of pieces of DNA, so that members of a gene family may end up on the wrong scaffold and therefore on the wrong linkage group, which need further to confirm.

### Conflict of interest statement

The authors declare that the research was conducted in the absence of any commercial or financial relationships that could be construed as a potential conflict of interest.
