# Supplementary figures and images for: The Malus domestica sugar transporter gene family: identifications based on genome and expression profiling related to the accumulation of fruit sugars
Source: Front Plant Sci. 2014 Nov 5;5:569. doi: 10.3389/fpls.2014.00569 (PMC4220645; doi:10.3389/fpls.2014.00569)

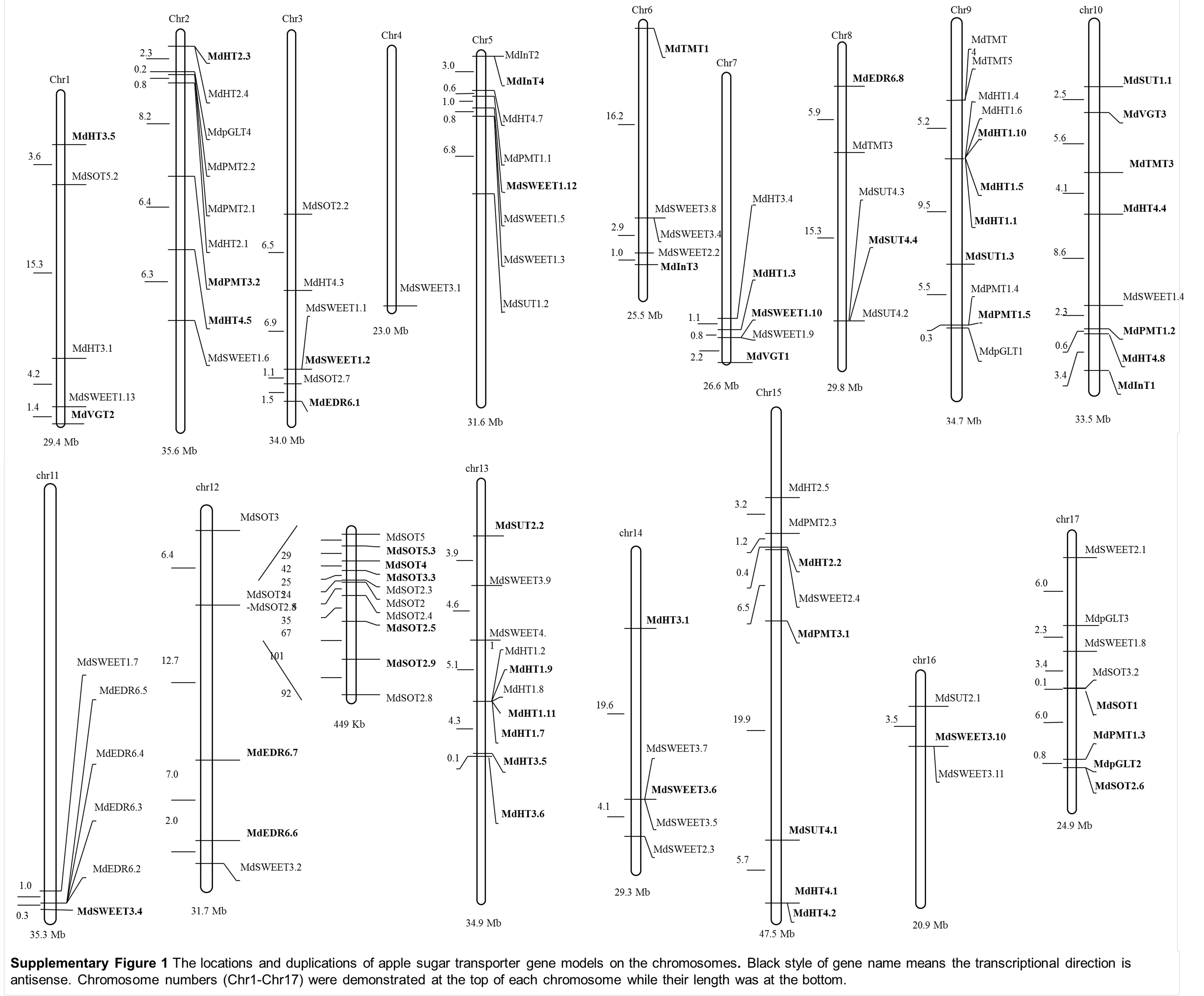

Supplement: Supplementary file 8 [file Image1.TIF]

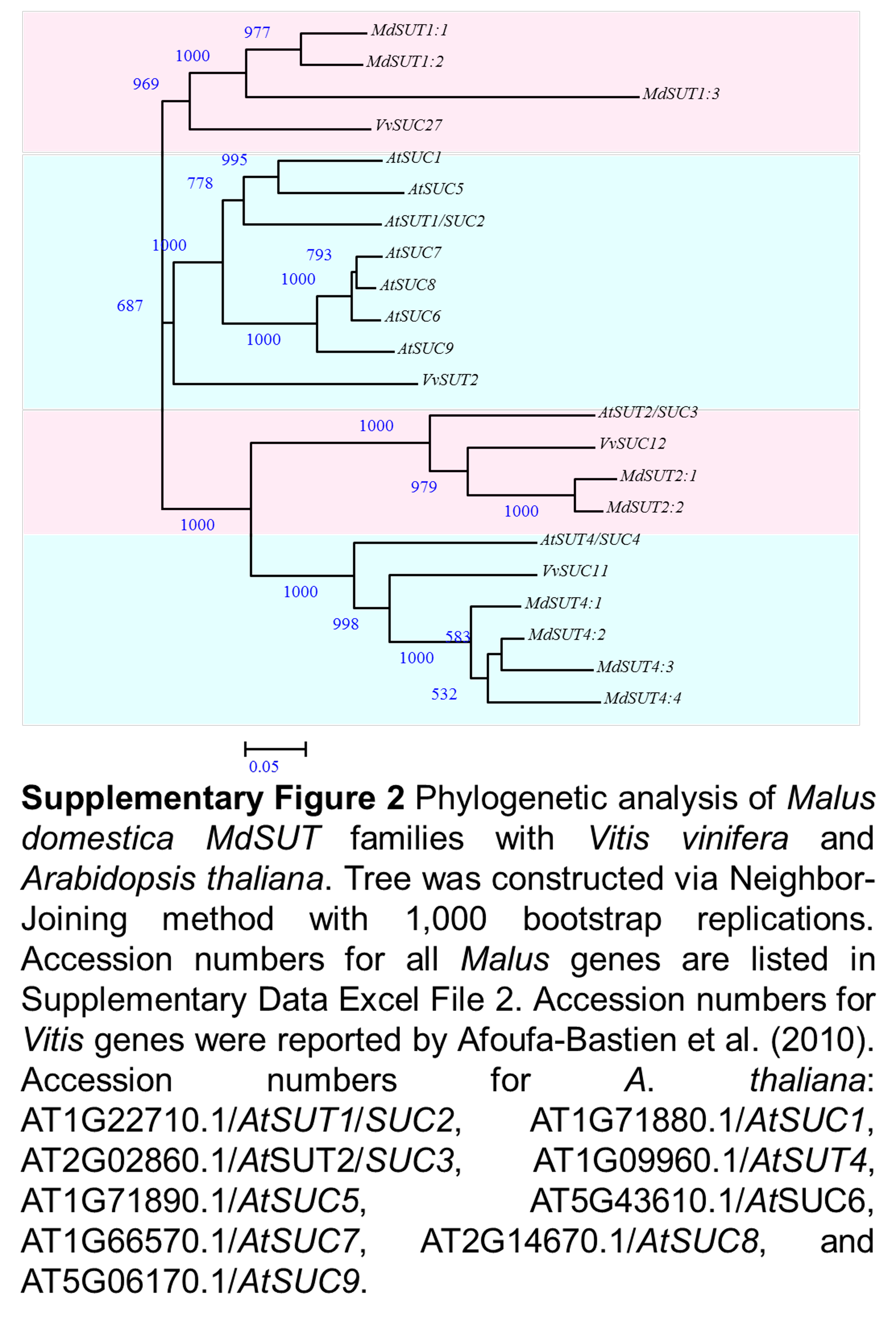

Supplement: Supplementary file 9 [file Image2.TIF]

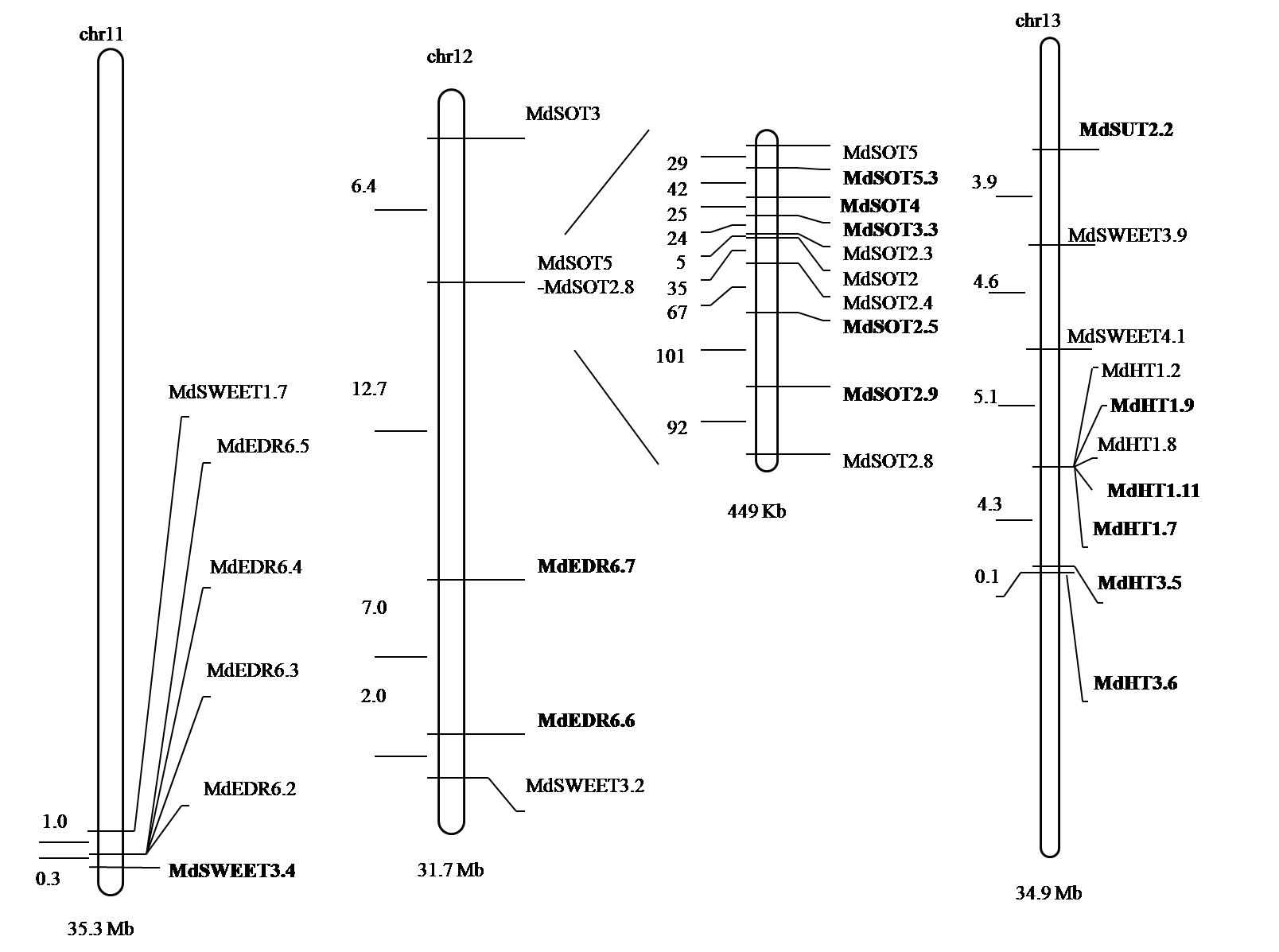

Supplement: Supplementary file 10 [file Image3.TIF]

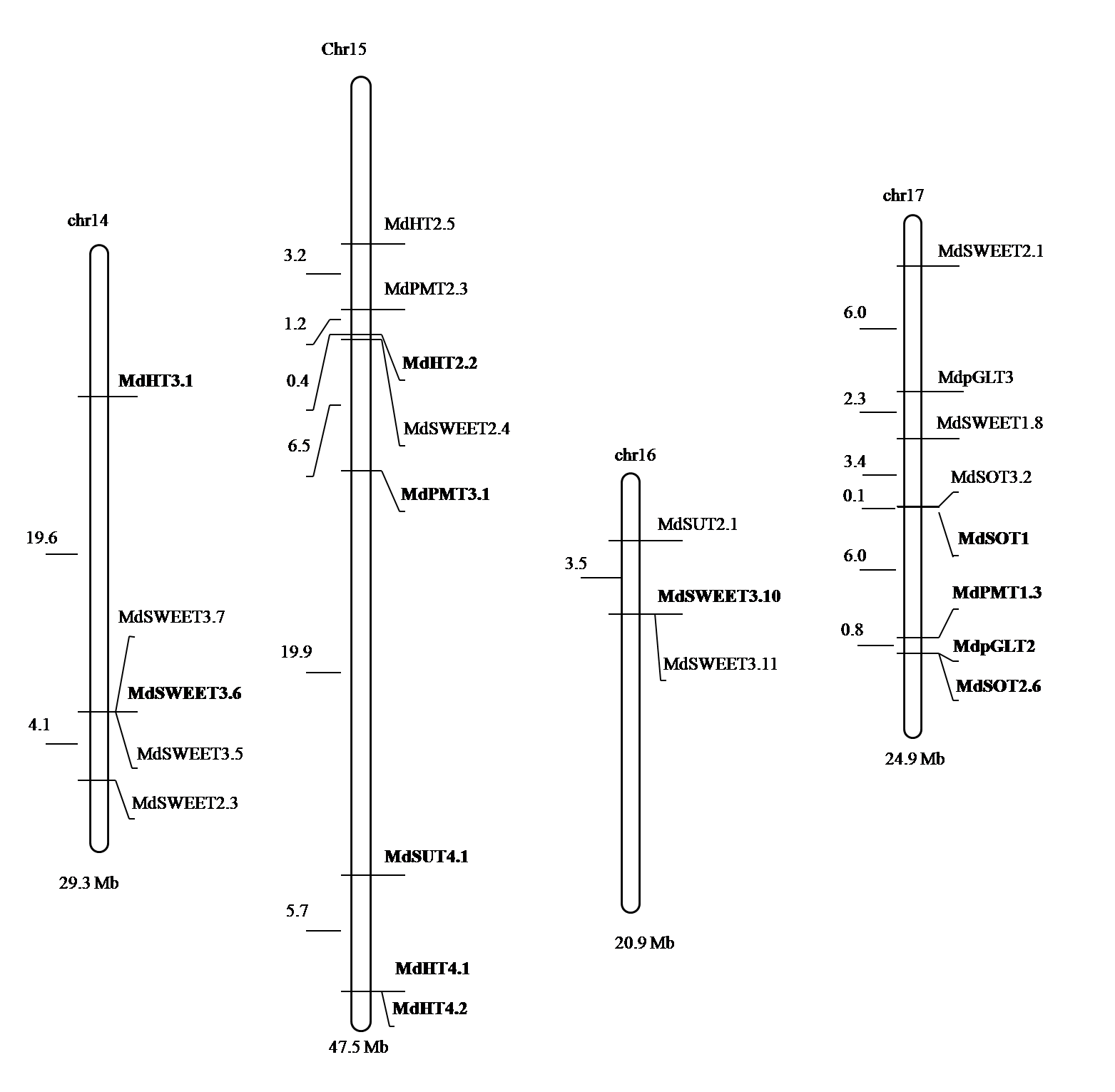

Supplement: Supplementary file 11 [file Image4.TIF]

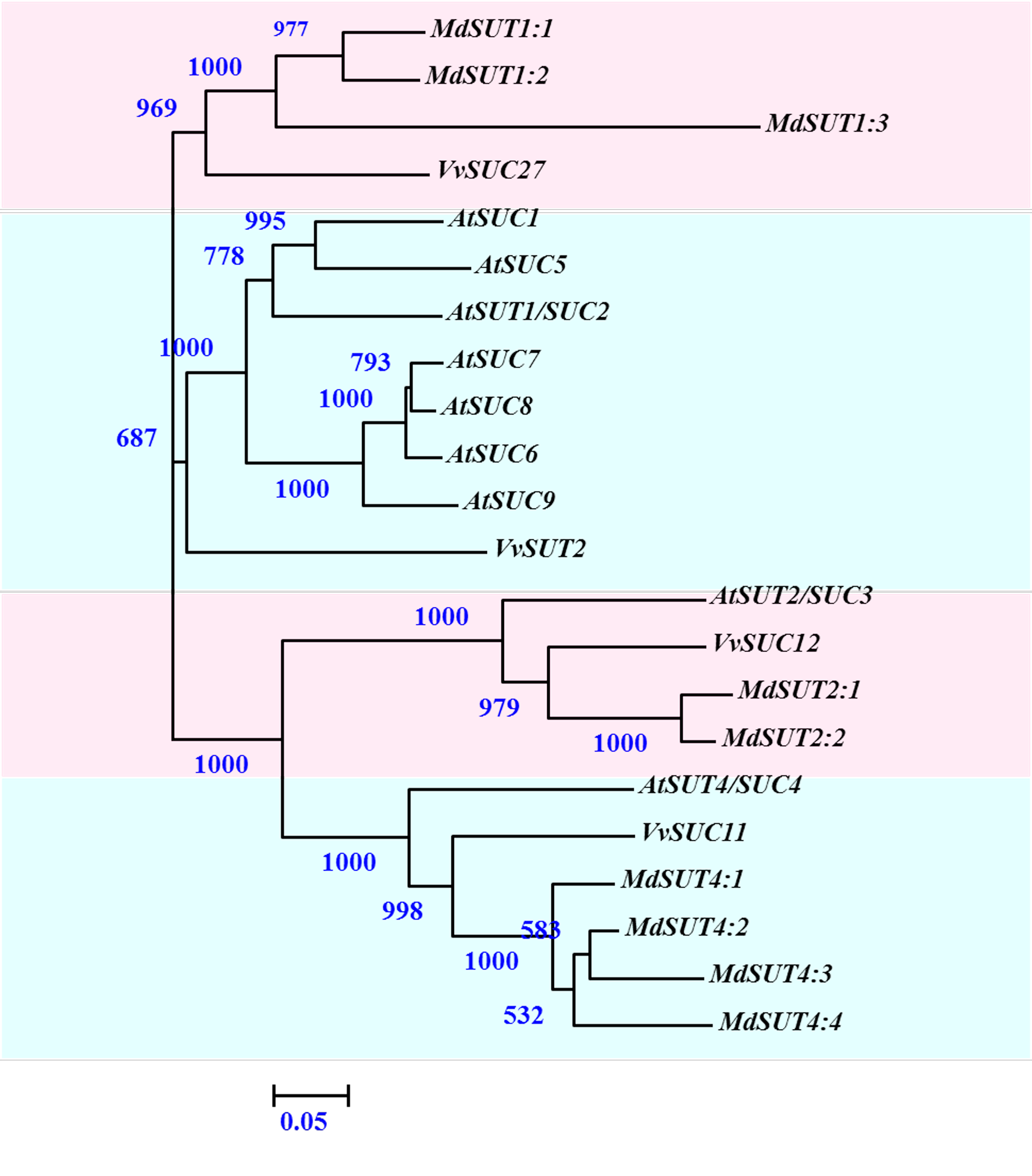

Supplement: Supplementary file 12 [file Image5.TIF]
